# A Genetic Selection for *dinB* Mutants Reveals an Interaction between DNA Polymerase IV and the Replicative Polymerase That Is Required for Translesion Synthesis

**DOI:** 10.1371/journal.pgen.1005507

**Published:** 2015-09-09

**Authors:** Michelle K. Scotland, Justin M. H. Heltzel, James E. Kath, Jung-Suk Choi, Anthony J. Berdis, Joseph J. Loparo, Mark D. Sutton

**Affiliations:** 1 Department of Biochemistry, School of Medicine and Biomedical Sciences, University at Buffalo, State University of New York, Buffalo, New York, United States of America; 2 Witebsky Center for Microbial Pathogenesis and Immunology, School of Medicine and Biomedical Sciences, University at Buffalo, State University of New York, Buffalo, New York, United States of America; 3 Department of Biological Chemistry and Molecular Pharmacology, Harvard Medical School, Boston, Massachusetts, United States of America; 4 Department of Chemistry, Cleveland State University, Cleveland, Ohio, United States of America; 5 The Center for Gene Regulation in Health and Disease, Cleveland State University, Cleveland, Ohio, United States of America; 6 Program in Genetics, Genomics and Bioinformatics, School of Medicine and Biomedical Sciences, University at Buffalo, State University of New York, Buffalo, New York, United States of America; Université Paris Descartes, INSERM U1001, FRANCE

## Abstract

Translesion DNA synthesis (TLS) by specialized DNA polymerases (Pols) is a conserved mechanism for tolerating replication blocking DNA lesions. The actions of TLS Pols are managed in part by ring-shaped sliding clamp proteins. In addition to catalyzing TLS, altered expression of TLS Pols impedes cellular growth. The goal of this study was to define the relationship between the physiological function of *Escherichia coli* Pol IV in TLS and its ability to impede growth when overproduced. To this end, 13 novel Pol IV mutants were identified that failed to impede growth. Subsequent analysis of these mutants suggest that overproduced levels of Pol IV inhibit *E*. *coli* growth by gaining inappropriate access to the replication fork *via* a Pol III-Pol IV switch that is mechanistically similar to that used under physiological conditions to coordinate Pol IV-catalyzed TLS with Pol III-catalyzed replication. Detailed analysis of one mutant, Pol IV-T120P, and two previously described Pol IV mutants impaired for interaction with either the rim (Pol IV^R^) or the cleft (Pol IV^C^) of the β sliding clamp revealed novel insights into the mechanism of the Pol III-Pol IV switch. Specifically, Pol IV-T120P retained complete catalytic activity *in vitro* but, like Pol IV^R^ and Pol IV^C^, failed to support Pol IV TLS function *in vivo*. Notably, the T120P mutation abrogated a biochemical interaction of Pol IV with Pol III that was required for Pol III-Pol IV switching. Taken together, these results support a model in which Pol III-Pol IV switching involves interaction of Pol IV with Pol III, as well as the β clamp rim and cleft. Moreover, they provide strong support for the view that Pol III-Pol IV switching represents a vitally important mechanism for regulating TLS *in vivo* by managing access of Pol IV to the DNA.

## Author Summary

Bacterial DNA polymerase IV (Pol IV) is capable of replicating damaged DNA *via* a process termed translesion DNA synthesis (TLS). Pol IV-mediated TLS can be accurate or error-prone, depending on the type of DNA damage. Errors made by Pol IV contribute to antibiotic resistance and adaptation of bacterial pathogens. In addition to catalyzing TLS, overproduction of *Escherichia coli* Pol IV impedes growth. In the current work, we demonstrate that both of these functions rely on the ability of Pol IV to bind the β sliding processivity clamp and switch places on DNA with the replicative Pol, Pol III. This switch requires that Pol IV contact both Pol III as well as two discrete sites on the β clamp protein. Taken together, these results provide a deeper understanding of how *E*. *coli* manages the actions of Pol III and Pol IV to coordinate high fidelity replication with potentially error-prone TLS.

## Introduction

Despite the actions of several DNA repair pathways, lesions capable of blocking progression of the replicative DNA polymerase (Pol) persist in the DNA template. Translesion DNA synthesis (TLS) represents one evolutionarily conserved mechanism by which organisms cope with these replication-blocking lesions [1–3]. In contrast to repair functions, which either reverse or excise the damage, TLS acts to bypass the damaged site using one or more specialized Pol, allowing the replication fork to proceed past the lesion [1]. Depending on the TLS Pol used, the DNA lesion and its sequence context, bypass may be either accurate or inaccurate [1–3]. Furthermore, due to the fact that most TLS Pols lack intrinsic proofreading activity and possess an open active site, these Pols display a significantly reduced fidelity when replicating undamaged DNA. Thus, TLS Pols may cause mutations by catalyzing inaccurate TLS, or by gaining inappropriate access to undamaged DNA during normal replication. A growing body of evidence supports the view that mutations introduced by TLS Pols contribute to antibiotic resistance and adaptation of microbial pathogens [4–7], as well as genome instability and cancer development in metazoans [8–11]. As a result, the actions of TLS Pols must be tightly regulated to limit unwanted mutations. Furthermore, TLS Pols replicate considerably slower than replicative Pols [12–14]. As a result, their unregulated access to the replication fork would significantly slow replication. The eukaryotic proliferating cell nuclear antigen (PCNA) and the bacterial β sliding clamp proteins play crucially important roles in managing the actions of TLS Pols, and in coordinating their activities with those of their respective replisomes *via* a process termed ‘Pol switching’ [12,15–20]. Pol-Pol interactions are also suggested to contribute to Pol switching [21–23]. However, several important questions regarding the mechanisms by which TLS Pols switch with replicative Pols, as well as the biological importance of the switching mechanism to regulation of TLS *in vivo* remain unanswered.

*E*. *coli* contains five distinct Pols, which are named Pols I-V. Pols II, IV and V act in TLS [1,3], while Pol I functions in DNA repair and Okazaki fragment maturation [24]. The 20-subunit Pol III holoenzyme serves as the bacterial replicase, and is composed of 2 homodimeric β sliding clamps and 3 core complexes (Pol IIIαεθ), which are tethered together *via* interaction of Pol IIIα with the heptameric DnaX ATPase (τ_3_δδ’ψχ) that acts to load the β clamp onto primed DNA [25,26]. The Pol IIIαεθ core complex performs DNA synthesis functions. Within this complex, Pol IIIα catalyzes DNA polymerization, Pol IIIε functions in proofreading and Pol IIIθ modestly stimulates Pol IIIε proofreading activity [25,26]. With the possible exception of Pol I, each of the 5 *E*. *coli* Pols contains either a pentameric (QLxLF) or hexameric (QLxLxL) clamp-binding motif (CBM) that is required for biological activity [27–31]. The CBM interacts with a hydrophobic cleft located near the C-terminus of each β clamp protomer. The different Pols also contact non-cleft surfaces of the β clamp, and these interactions likewise contribute to Pol function and/or Pol switching [17,32–37]. To date, structural information regarding these non-cleft contacts is restricted to Pol IV. In the co-crystal structure of the complex consisting of the Pol IV little finger domain (Pol IV^LF^; residues 243–351) bound to the β clamp, residues ^303^VWP^305^ of Pol IV interacted with positions E93 and L98 on the rim of the β clamp, while the C-terminal hexameric CBM (^346^QLVLGL^351^) of Pol IV extended over the β clamp dimer interface to interact with the β clamp cleft of the adjacent β clamp protomer [32]. Using a primer extension assay, we previously demonstrated that while only the Pol IV-β clamp cleft interaction was required for processive replication, both the β clamp rim and cleft interactions contributed to Pol III-Pol IV switching *in vitro* [12,17,38]. In contrast to our findings, Gabbai and colleagues, utilizing a different assay that may more accurately represent the structure and composition of the replisome, concluded that the Pol IV-β clamp rim contact stimulated but was not required for a Pol III-Pol IV switch *in vitro* [18]. In light of this finding, they suggested direct competition between Pol III and Pol IV for the β clamp cleft as an alternative mechanism for their switching. In addition to switching, Pol IV can be recruited directly to single strand (ss) DNA gaps generated by Pol III skipping over lesions in the template strand to continue replication downstream of the block [1,39]. In summary, recruitment of TLS Pols to lesions is suggested to occur by two different mechanisms: (i) β clamp may recruit TLS Pols post-replicatively to lesions within ssDNA DNA gaps generated by Pol III skipping, or (ii) TLS Pols may be recruited to the replication fork and access lesions after undergoing a switch with Pol III. However, the extent to which these proposed mechanisms are utilized *in vivo* has not yet been determined. Furthermore, the biological relevance of the Pol IV-β clamp rim interaction to the TLS function of Pol IV is also unknown.

*E*. *coli* Pol IV catalyzes accurate bypass of *N* ^*2*^-dG lesions induced by nitrofurazone (NFZ), as well as alkylated adducts such as *N* ^*3*^-methyladenine (*N* ^*3*^-mdA) caused by methyl methanesulfonate (MMS). As a result, *E*. *coli* strains lacking Pol IV function (*i*.*e*., Δ*dinB*) display sensitivity to these agents [17,40–43]. In contrast to this protective role, overproduction of Pol IV is lethal to *E*. *coli* [34,44,45]; similarly, aberrant expression of Pol κ, the eukaryotic ortholog of *dinB*, promotes genome instability in human cells [46]. Lethality in aerobically cultured *E*. *coli* cells was suggested to result from toxic levels of double strand (ds) DNA breaks resulting from efforts to repair closely spaced 8-oxo-7,8-deoxyguanosine (8-oxo-dG) adducts incorporated during replication of undamaged DNA by Pol IV [47]. However, sensitivity of a *dnaN159* strain to ~4-fold higher than SOS-induced levels of Pol IV [34,44], and of a *dnaN* ^*+*^ *E*. *coli* strain to significantly higher than SOS-induced levels (*i*.*e*., ~70-fold; [45]), were both independent of Pol IV catalytic activity, suggesting that at least in these cases, lethality relied on one or more alternative mechanisms. The *dnaN159* allele encodes a mutant form of the β sliding clamp that is deficient in regulation of proper access of the different *E*. *coli* Pols to DNA [34,35,38,44,48,49]. Thus, sensitivity of the *dnaN159* strain to elevated levels of Pol IV was suggested to result from its enhanced ability to replace the bacterial Pol III replicase at the replication fork, thereby disrupting DNA replication [34,44,48]. Consistent with a Pol III-Pol IV switch underlying this lethal phenotype, mutations in Pol IV that disrupt its ability to interact with either the cleft (Pol IV^C^; see Table 1) or the rim (Pol IV^R^) of the β clamp abrogated its ability to kill the *dnaN159* strain [38]. Finally, SOS-induced levels of Pol IV modestly slowed the rate of DNA replication *in vitro* [13,14,45], while overproduction of Pol IV severely impeded it, possibly by replacing Pol III [13,14,45]. The finding that Pol IV^LF^-β clamp interactions were dispensable when Pol IV was expressed at ~70-fold higher than SOS-induced levels suggested that Pol IV may interact with Pol III to effect its displacement from the β clamp [45]. Based on these results, Pol IV was suggested to act as a DNA damage checkpoint effector that acts to slow replication fork progression in response to DNA damage [13,45].

**Table 1:** Bacterial strains and plasmid DNAs used in this study.

***E*. *coli* strains**		
**Strain**	**Relevant genotype**	**Source** ^*a*^
DH5α	ϕ80*lacZ*ΔM15 Δ(*lacZYA-argF*)*U169 recA1 endA1 hsdR17*(r_K_^−^m_K_^+^) *phoA supE44 thi*-1 *gyrA96 relA1*	NEB
RW118	*thr*-1 *araD139* Δ(*gpt-proA*)*62 lacY1 tsx*-33 *supE44 galK2 hisG4*(Oc) *rpsL31 xyl*-5 *mtl*-1 *argE3*(Oc) *thi*-1 *sulA211*	[85]
MS100	RW118: *dnaN* ^*+*^ *tnaA300*::Tn*10*	[48]
MS104	RW118: *dnaN* ^*+*^ *tnaA300*::Tn*10 lexA51*(Def)	[48]
MS105	RW118: *dnaN159 tnaA300*::Tn*10 lexA51*(Def)	[48]
MS116	RW118: *dnaN159 tnaA300*::Tn*10* Δ*uvrB*::*cat* Δ(*dinB*-*yafN*)::*kan*	[48]
RW120	RW118: Δ*umuDC595*::*cat*	[85]
VB102	RW118: Δ(*dinB-yafN*)::*kan*	This work
VB103	RW118: Δ(*dinB-yafN*)::*kan* Δ*umuDC595*::*cat*	This work
JH200	RW118: Δ*uvrB*::*cat* Δ(*dinB*-*yafN*)::*kan*	This work
CC108	*ara* Δ(*lac-pro*) (F *lacI373 lacZ*[6G→5G] *proB* ^*+*^)	[51]
JW0097	Δ(*araD-araB*)*567* Δ*mutT790*::*kan* Δ*lacZ4787*(::*rrnB*-3) *rph-*1 Δ(*rhaD-rhaB*)*568 hsdR514*	CGSC
MG1655	*rph-1*	CGSC
MKS100	MG1655: Δ*dinB749*::*kan*	This work
MKS101	MG1655: Δ*umuDC595*::*cat*	This work
MKS102	MG1655: Δ*dinB749*::*kan* Δ*umuDC595*::*cat*	This work
MKS103	MG1655: *zaf-3633*::*cat dinB* ^*+*^ (Pol IV^+^)	This work
MKS104	MG1655: *zaf-3633*::*cat dinB80* (Pol IV-D103N)	This work
MKS105	MG1655: *zaf-3633*::*cat dinB81* (*dinB*Δ*347–351*; Pol IV^C^)	This work
MKS106	MG1655: *zaf-3633*::*cat dinB82* (*dinB-V303A-W304G-P305A*; Pol IV^R^)	This work
MKS107	MG1655: *zaf-3633*::*cat dinB89* (Pol IV-T120P)	This work
BL21(DE3)	*dcm ompT hsdS*(r_B_^−^m_B_^–^) *gal* (*malB* ^*+*^)_K-12_ λDE3	Novagen
BL21(DE3)Δ*dinB*	BL21(DE3): Δ(*dinB-yafN*)::*kan*	This work
**Plasmid DNAs**		
**Plasmid**	**Relevant characteristics**	**Source**
pWSK29	Amp^R^; pSC101 origin	[86]
pRM102	Amp^R^; pWSK29 bearing *dinB* ^*+*^ (Pol IV^+^)	[44]
pJH110	Amp^R^; pRM102 containing an NdeI site overlapping the N-terminal methionine of *dinB* ^*+*^ (Pol IV^+^)	This work
pJH101	Amp^R^; pWSK29 bearing *dinB-V303A/W304G/P305A* (Pol IV^R^)	[17]
pJH102	Amp^R^; pWSK29 bearing *dinB*Δ*347351* (Pol IV^C^)	[17]
pJH100	Amp^R^; pWSK29 bearing *dinB-D103N* (Pol IV-D103N)	[17]
pET11a	Amp^R^; ColE1 origin and T7 promoter	Novagen
pRM112	Amp^R^; pET11a bearing *dinB* ^*+*^ (Pol IV^+^)	[34]
pRM112-D103N	Amp^R^; pET11a bearing *dinB80* (Pol IV-D103N)	[17]
pRM112-C	Amp^R^; pET11a bearing *dinB81* (Pol IV^C^)	[17]
pRM112-R	Amp^R^; pET11a bearing *dinB82* (Pol IV^R^)	[17]
pRM112-D10G	Amp^R^; pET11a bearing *dinB-D10G* (Pol IV-D10G)	This work
pRM112-A15V	Amp^R^; pET11a bearing *dinB-A15V* (Pol IV-A15V)	This work
pRM112-A44D	Amp^R^; pET11a bearing *dinB-A44D* (Pol IV-A44D)	This work
pRM112-G52V	Amp^R^; pET11a bearing *dinB-G52V* (Pol IV-G52V)	This work
pRM112-C66S	Amp^R^; pET11a bearing *dinB-C66S* (Pol IV-C66S)	This work
pRM112-R75L	Amp^R^; pET11a bearing *dinB-R75L* (Pol IV-R75L)	This work
pRM112-T120P	Amp^R^; pET11a bearing *dinB-T120P* (Pol IV-T120P)	This work
pRM112-A143E	Amp^R^; pET11a bearing *dinB-A143E* (Pol IV-A143E)	This work
pRM112-A149D	Amp^R^; pET11a bearing *dinB-A149D* (Pol IV-A149D)	This work
pRM112-G183V	Amp^R^; pET11a bearing *dinB-G183V* (Pol IV-G183V)	This work
pRM112-G219V	Amp^R^; pET11a bearing *dinB-G219V* (Pol IV-G219V)	This work
pRM112-H302Q/Q342K	Amp^R^; pET11a bearing *dinB-H302Q*/Q342K (Pol IV-H302Q/Q342K)	This work
pRM112-R323S	Amp^R^; pET11a bearing *dinB-R323S* (Pol IV-R323S)	This work
pBAD	Amp^R^; ColE1 origin and *araBAD* promoter control	Invitrogen
pDB10	Amp^R^; pBAD-HisA bearing *dinB* ^*+*^ (Pol IV^+^)	[45]
pDB33	Amp^R^; pDB10 bearing *dinB-T120P* (Pol IV-T120P)	This work
pDB14	Amp^R^; pBAD-HisA bearing *dinB* residues 231–351 (Pol IV^LF^)	[45]
pDB12	Amp^R^; pBAD-HisA bearing *dinB* residues 1–230 (Pol IV^CD^)	[45]
pDB20	Amp^R^; pDB12 bearing *dinB* ^*CD*^ *-D10G* (Pol IV^CD^-D10G)	This work
pDB21	Amp^R^; pDB12 bearing *dinB* ^*CD*^ *-C66S* (Pol IV^CD^-C66S)	This work
pDB22	Amp^R^; pDB12 bearing *dinB* ^*CD*^ *-R75L* (Pol IV^CD^-R75L)	This work
pDB23	Amp^R^; pDB12 bearing *dinB* ^*CD*^ *-T120P* (Pol IV^CD^-T120P)	This work
pDB25	Amp^R^; pDB12 bearing *dinB* ^*CD*^ *-G183V* (Pol IV^CD^-G183V)	This work
pKD46	Amp^R^; expresses λRed from *araBAD* promoter	[74]
pGEM-T	Amp^R^; cloning vector	Promega
pMKS100	Amp^R^; pGEM-T bearing *lafU’ zaf-3633*::*cat dinB* ^*+*^ (Pol IV^+^) *yafN’*	This work
pMKS101	Amp^R^; pGEM-T bearing *lafU’ zaf-3633*::*cat dinB80* (Pol IV-D103N) *yafN’*	This work
pMKS102	Amp^R^; pGEM-T bearing *lafU’ zaf-3633*::*cat dinB81* (Pol IV^C^) *yafN’*	This work
pMKS103	Amp^R^; pGEM-T bearing *lafU’ zaf-3633*::*cat dinB82* (Pol IV^R^) *yafN’*	This work
pMKS104	Amp^R^; pGEM-T bearing *lafU’ zaf-3633*::*cat dinB89* (Pol IV-T120P) *yafN’*	This work

^***a***^ See *Materials and Methods* for a description of how strains and plasmid DNAs were constructed and verified.

NEB, New England Biolabs; CGSC, *E*. *coli* Genetic Stock Center, Yale University.

The goal of this study was to define the relationship between the physiological function of Pol IV in TLS and its ability to impede *E*. *coli* growth when overproduced. With this goal in mind, the hypersensitivity of the *dnaN159* strain was exploited to identify 13 novel mutant Pol IV proteins that failed to confer lethality. Genetic and biochemical characterization of these Pol IV mutants strongly suggest that the ability of overproduced levels of Pol IV to inhibit *E*. *coli* growth is a consequence of its ability to gain inappropriate access to the replication fork *via* a switch that is mechanistically similar to that used under physiological conditions to coordinate the actions of Pol IV with Pol III. Importantly, further analysis of one of the mutants, Pol IV-T120P, revealed novel insights into the mechanism by which Pol IV gains access to DNA lesions *in vivo*. Specifically, Pol IV-T120P retained complete catalytic activity *in vitro* but, like Pol IV^R^ and Pol IV^C^, failed to support Pol IV TLS function *in vivo*. Using a single molecule primer extension assay, we demonstrated that the T120P mutation abrogated a biochemical interaction of Pol IV with Pol III that was required for Pol III-Pol IV switching. Taken together, these results suggest that Pol III-Pol IV switching involves interaction of Pol IV with both Pol III and the β clamp rim and cleft regions, and provide strong support for the view that Pol III-Pol IV switching represents a vitally important mechanism for regulating TLS *in vivo* by managing access of Pol IV to the DNA.

## Results

### Identification of novel Pol IV mutations that fail to impede growth of the *dnaN159 E*. *coli* strain

Based on results of a quantitative transformation assay, the hypersensitivity of the *dnaN159 lexA51*(Def) strain (MS105) to ~4-fold higher than SOS-induced levels of Pol IV expressed from its native LexA-regulated promoter present in pRM102 was independent of Pol IV catalytic activity ([38]; see Pol IV-D103N in S1 Table). Moreover, the ability of Pol IV to impede growth of MS105 was independent of dsDNA breaks stemming from the incorporation into nascent DNA of oxidized precursors, since lethality was observed regardless of whether MS105 was grown aerobically or anaerobically (S2 Table). In contrast, lethality of the *dnaN159* strain required the ability of Pol IV to interact with both the rim and cleft of the β clamp ([38]; see Pol IV^R^ and Pol IV^C^ in S1 Table). Taken together, these results suggest that lethality was caused by inappropriate access of Pol IV to the replication fork, rather than its ability to incorporate oxidized precursors into nascent DNA [47]. With the goal of gaining insight into the mechanism by which elevated levels of Pol IV impeded growth, a genetic assay was used to select for novel Pol IV mutants that are unable to kill the *dnaN159* strain. From a total of ~2 x 10^7^ independent clones, 16 plasmid-encoded *dinB* mutants expressing a full length Pol IV protein were identified (S1 Fig). These mutants corresponded to 13 unique *dinB* alleles: 12 contained a single missense mutation, while one contained two missense mutations (Table 2). Using our quantitative transformation assay referred to above [38], we confirmed that each of these mutant Pol IV-expressing plasmids was unable to impede growth of the *dnaN159* strain (S1 Table). For comparison, the wild type Pol IV-expressing plasmid pRM102 was more than 1,000-fold less efficient at transforming MS105 relative to the pWSK29 control (S1 Table).

**Table 2:** Summary of novel *dinB* alleles.

Plasmid	Nucleotide substitution(s) ^*a*^	Deduced amino acid substitution(s)	*dinB* allele ^*b*^	Occurrences ^*c*^	Proposed function ^*d*^	Overproduction phenotype ^*e*^
pJH111	^28^GAC^30^→GGC	D10G	*dinB83*	1	Structural integrity	+
pJH150	^43^GCA^45^→GTA	A15V	*dinB84*	1	Structural integrity	−
pJH152	^130^GCC^132^→GAC	A44D ^*f*^	*dinB85*	3	Unknown	−
pJH154	^154^GGC^156^→GTC	G52V	*dinB86*	1	Hydrophobic	−
	^937^GGG^939^→GGC	G313 (silent) ^*g*^			core	
pJH112	^196^TGC^198^→AGC	C66S	*dinB87*	2	Hydrophobic core	+
pJH113	^223^CGC^225^→CTC	R75L	*dinB88*	1	Tertiary structure	+/− ^*h*^
pJH114	^358^ACC^360^→CCC	T120P	*dinB89*	1	Unknown	+
pJH156	^427^GCA^429^→GAA	A143E	*dinB90*	1	Hydrophobic core	−
pJH115	^445^GCC^447^→GAC	A149D	*dinB91*	1	Unknown	−
pJH116	^547^GGC^549^→GTC	G183V	*dinB92*	1	DNA interaction	+
pJH157	^655^GGC^657^→GTC	G219V	*dinB93*	1	DNA interaction	−
pJH117	^904^CAC^906^→CAA	H302Q	*dinB94*	1	β clamp	+
	^1024^CAA^1026^→AAA	Q342K			interaction	
pJH118	^967^CGC^969^→AGC	R323S	*dinB95*	1	DNA interaction	+

^***a***^ Nucleotide changes are represented in the context of the codon in which they reside. Numbers refer to the first and last base in the codon, with position 1 representing the A in the ATG encoding the first amino acid of Pol IV (*i*.*e*., ^1^ATG^3^).

^***b***^ Allele numbers were assigned by the *E*. *coli* Genetic Stock Center (CGSC) at Yale.

^***c***^ The number of times that each mutation was identified is indicated.

^***d***^ Proposed functions for Pol IV residues are based largely on structural insights from Bunting *et al*. [32], Sharma *et al*. [53], Boudsocq *et al*. [54] and Ling *et al*. [55].

^***e***^ When expressed at high levels from a T7 promoter, recombinant Pol IV mutant proteins were either soluble (+), or poorly soluble and/or extensively proteolyzed (–), as indicated.

^***f***^ One of the three A44D clones identified contained an additional silent mutation at I74 (^220^ATC^222^→^220^ATT^222^). This allele was not further characterized.

^***g***^ The ^937^GGG^939^→GGC substitution present in plasmid pJH154 represents a silent mutation at position G313.

^***h***^ The Pol IV-R75L mutant was expressed as a soluble full-length protein from the T7 promoter, but was inconsistently detected following expression in Pol IV^CD^ from the arabinose promoter of plasmid pDB22, suggesting Pol IV^CD^-R75L was misfolded.

In addition to impeding *E*. *coli* growth, overproduced levels of Pol IV also promote –1 frameshift mutations within homopolymeric runs of dG or dA [50], while SOS-induced levels confer UV sensitivity upon the *dnaN159* strain [38,48]. Since these phenotypes appear to result from the ability of Pol IV to gain inappropriate access to the replication fork [34,44,48], we hypothesized that the Pol IV mutations described above would likewise be impaired for these functions. Based on results of a *lacZ* ^*–*^→LacZ^+^ reversion assay [51,52], all 13 mutant *dinB* alleles were impaired for promoting –1 frameshift mutations (S2 Fig). Likewise, these mutants were also impaired for conferring UV sensitivity upon the *dnaN159* strain (S3 Fig). Taken together, these results suggest that the mutations identified in Pol IV that prevented it from killing the *dnaN159* strain served to impair its catalytic activity and/or its ability to gain access to the DNA template *in vivo*.

**Fig 1:**
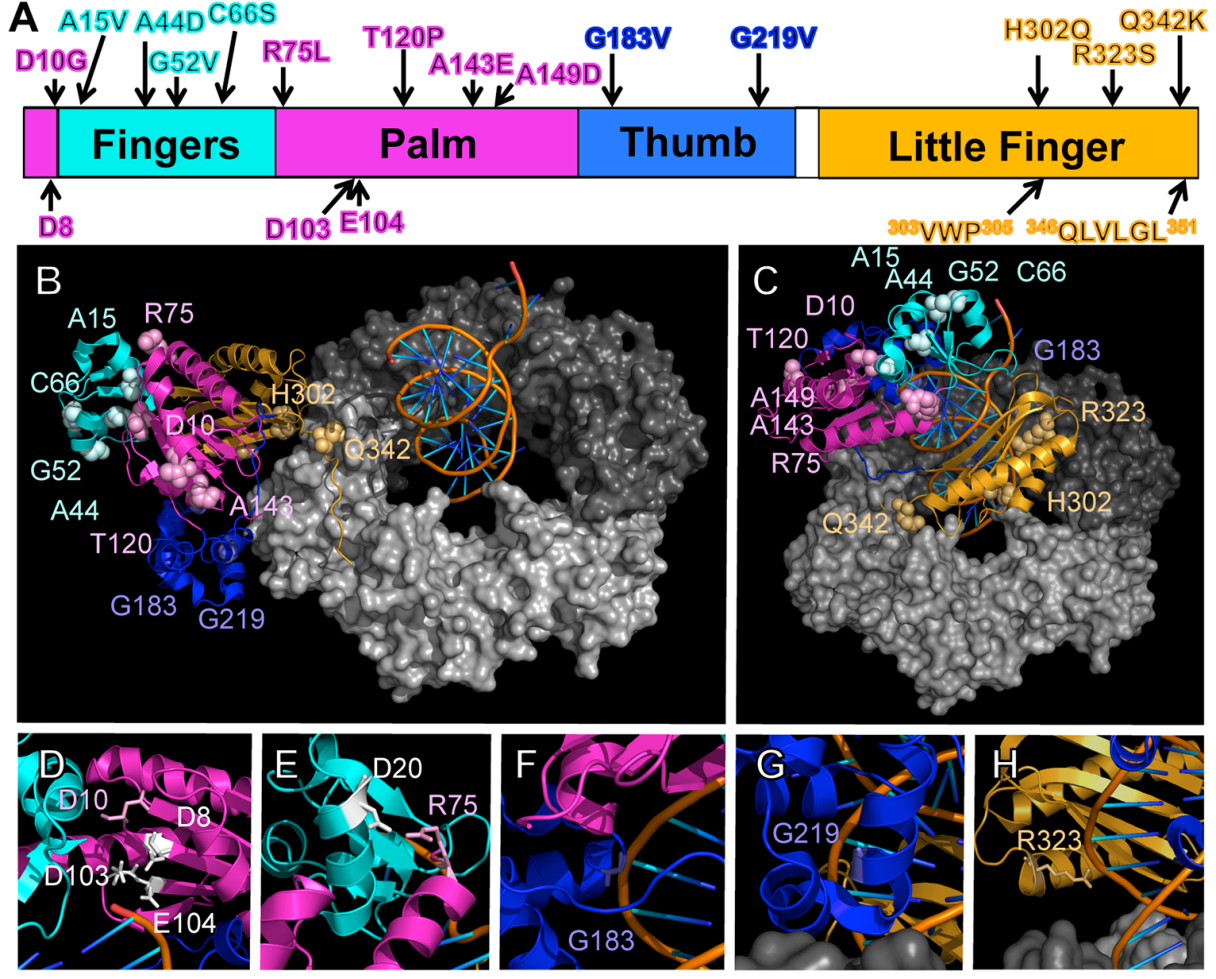
Positions of mutations represented on structural models for the Pol IV-β clamp-DNA complex. **(A)** Positions of the mutations identified in this work are represented on a linear model of Pol IV. Each domain of Pol IV is color-coded: palm domain (residues 1–10 and 74–165), magenta; fingers domain (residues 11–73), cyan; thumb domain (residues 166–240), blue; little finger domain (residues 243–351), orange. Residues involved in Pol IV catalytic activity (D8, D103, and E104), and β clamp rim (^303^VWP^305^) and β cleft (^346^QLVLGL^351^) interactions are highlighted on the bottom of the linear diagram. Pymol models of β clamp assembled on DNA and bound to Pol IV in either a **(B)** non-replicative or **(C)** replicative mode. In the non-replicative mode, Pol IV^LF^ contacts both the rim and the cleft of the β clamp. Interaction of the Pol IV^LF^ with the β clamp rim acts to pull Pol IV to the side of the β clamp and away from the DNA template. In the replicative mode, Pol IV is bound to the β clamp cleft and the DNA template. Since it is no longer associated with the β clamp rim, Pol IV sits on the face of the β clamp and can now access the DNA template. These models were built using MacPyMol Molecular Graphics System, Version 1.7.4 Schrödinger, LLC and coordinates for the Pol IV-DNA (PDB 4IR9) and Pol IV^LF^ domain in complex with β clamp (PDB 1UNN). For the model depicting the non-replicative mode of binding, the Pol IV^LF^ domain of Pol IV in the Pol IV-DNA structure was aligned with the Pol IV^LF^ domain in the Pol IV^LF^-β clamp complex. For the replicative mode, Pol IV was rotated to the face of the β clamp by aligning it with the DNA template passing though the center of β clamp while maintaining the interaction of Pol IV^LF^ with the β clamp cleft. Once full length Pol IV was aligned, the Pol IV^LF^ structure in 1UNN was hidden from view. 4IR9 does not include residues 342–351; hence residues 342–351 in 1UNN were left visible to complete the structure of Pol IV in both models. **(D)** The position of residue D8 relative to D10, D103 and E104, which comprise the catalytic center of Pol IV, is shown, as are **(E)** residues R75 and D20, which may form a hydrogen bond between the palm and the fingers domain, helping to stabilize the tertiary structure of Pol IV, and residues **(F)** G183, **(G)** G219 and **(H)** G323, which may contact the DNA template.

As summarized in Fig 1A, the identified Pol IV mutations are distributed throughout all 4 structural domains of Pol IV. To gain insight into the possible defect(s) associated with each mutant Pol IV, the positions of the mutations identified in each of the 13 *dinB* alleles were represented on *in silico* models for the structure of the Pol IV-β clamp complex assembled on primed DNA in either a non-replicative (meaning Pol IV is bound to both the β clamp rim and cleft, but not the DNA; see Fig 1B) or a replicative mode (meaning Pol IV is bound only to the β clamp cleft, as well as the DNA; see Fig 1C). Based upon these structural models, combined with our current understanding of *E*. *coli* Pol IV structure-function [32,53], as well as published studies of the homologous *Sulfolobus solfataricus* P2 Dpo4 [54,55], we inferred likely functions for several of the mutated residues in Pol IV (Table 2). Residues A15, G52, C66 and A143 likely contribute to either structural integrity or the hydrophobic core (Fig 1A–1C), suggesting that mutations of these residues most likely alter overall Pol IV structure. Position D10 likely contributes to structural integrity and is adjacent to D8, D103 and E104 (Fig 1D), which together act to coordinate 2 Mg^+2^ ions to constitute the catalytic core of Pol IV, suggesting that substitution of D10 with glycine might affect the structure of the Pol IV catalytic center. Residue R75 resides in the fingers domain and appears to form a hydrogen bond with residue D20 in the palm (Fig 1E), possibly contributing to the tertiary structure of Pol IV. Residues G183, G219, and R323 are all in close proximity to the DNA template and may be involved in Pol IV-DNA interactions (Fig 1F–1H). Finally, H302 and Q342 of Pol IV are immediately adjacent to residues previously demonstrated by Bunting *et al*. to directly contact the β clamp rim (^303^VWP^305^) or cleft (^346^QLVLGL^351^), respectively, suggesting that the H302Q and Q342K substitutions disrupt these interactions ([32]; Fig 1B). While presumed functions could be assigned for many of the mutants based upon previous studies, possible defects of the Pol IV mutants bearing substitutions of residues A44, T120 or A149 remain unclear (Table 2).

### Overexpression of Pol IV catalytic domain mutants fails to impede growth of *dnaN* ^*+*^ *E*. *coli*

**Fig 2:**
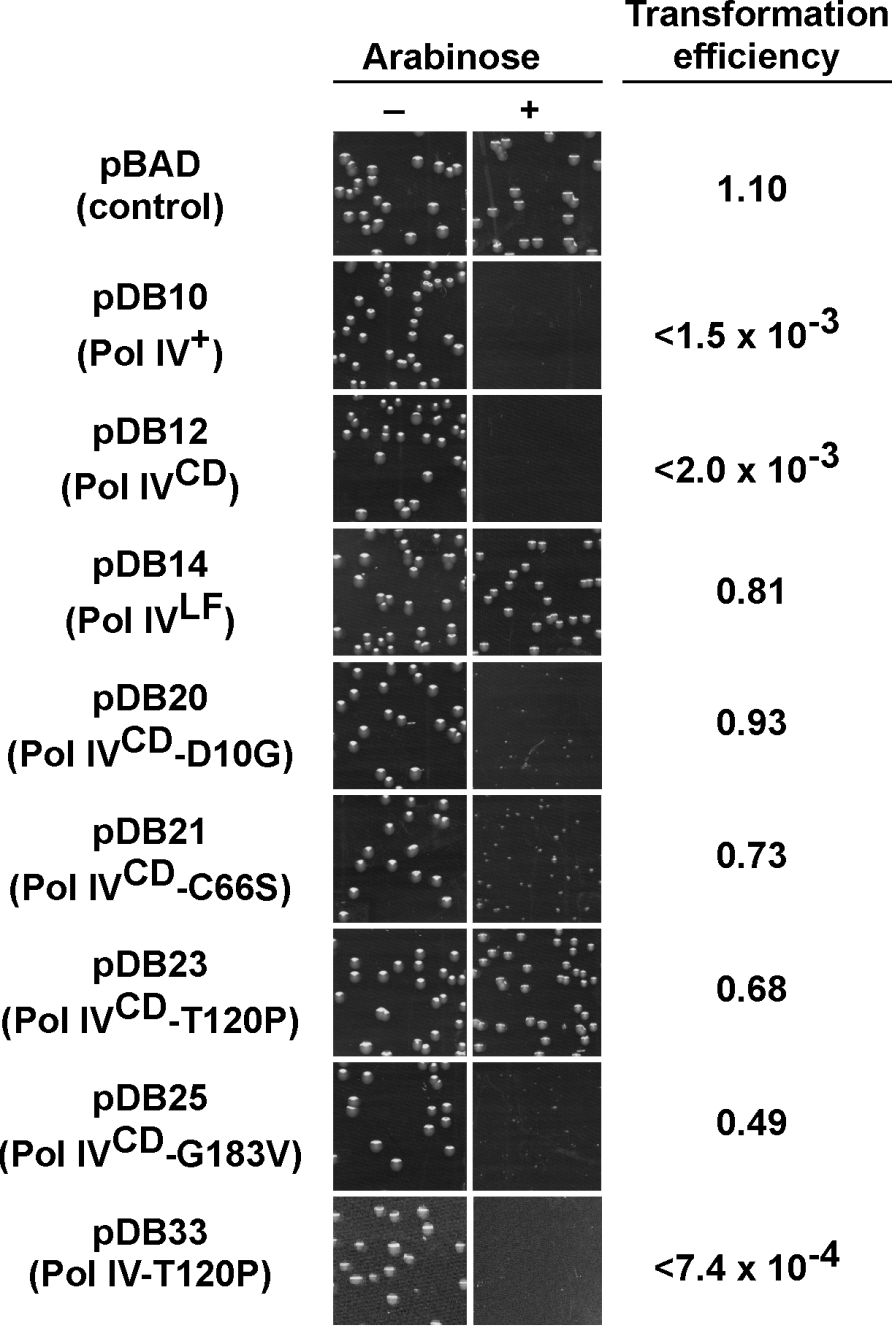
Ability of mutations in Pol IV^CD^ to impede *E*. *coli* growth when overexpressed. *E*. *coli* strain MS100 was transformed with the indicated plasmids, and aliquots of each transformation reaction were spread onto M9 minimal media supplemented with either glucose or arabinose, as noted. Representative portions of each plate following overnight incubation at 30°C are shown, as well as the ratio of the transformation frequency observed on plates supplemented with arabinose divided by the frequency observed on plates lacking arabinose. Results shown are representative of 2 separate experiments. The steady state level of Pol IV^CD^ and Pol IV^CD^-T120P were measured in soluble cell free protein extracts by densitometry of Coomassie Brilliant Blue stained SDS-PAGE following arabinose induction. Based on the density of the region encompassing Pol IV^CD^, minus the background density observed in the pBAD control, wild type Pol IV^CD^ (7.06±1.55 density units) and Pol IV^CD^-T120P (7.76±1.09 density units) were present at comparable levels.

In order to gain insight into the relationship between the abilities of elevated levels of Pol IV to impede growth of the *dnaN159* strain [34,38,44] and overproduced levels of Pol IV to kill the *dnaN* ^+^ strain [45], a quantitative transformation assay was used to analyze the phenotypes of pBAD derivatives bearing the relevant Pol IV mutations (see Table 1). The ability of overproduced levels of full-length Pol IV (pDB10) or the catalytic domain of Pol IV (Pol IV^CD^; residues 1–230 expressed from pDB12) to impede growth of *E*. *coli* was independent of both its catalytic activity [45] and aerobic growth (S4 Fig), similar to the situation discussed above for the *dnaN159* strain (S1 Table and S2 Table). Since overexpression of Pol IV^CD^ was necessary and sufficient to impede growth ([45]; see Fig 2), we focused on Pol IV mutations mapping within the first 230 residues of Pol IV. Despite the fact that the mutant Pol IV proteins appeared stable when expressed from their native *dinB* promoter contained within a low copy number plasmid (S1 Fig), 6 of the 11 mutants containing substitutions within the first 230 residues of Pol IV displayed either poor solubility or signs of extensive proteolysis following their overproduction from the T7 promoter while one mutant (R75L) was specifically unstable when cloned into Pol IV^CD^-expressing pBAD plasmid (see Table 2). These Pol IV mutants were not pursued further. Results for the other 4 Pol IV mutants are summarized in Fig 2. The plasmids overproducing Pol IV^CD^-D10G (pDB20), Pol IV^CD^-C66S (pDB21), Pol IV^CD^-T120P (pDB23), or Pol IV^CD^-G183V (pDB25) each transformed the *dnaN* ^*+*^ strain with an efficiency comparable to that of the pBAD control, both in the presence or absence of arabinose (albeit most exhibited tiny to small colonies), while the plasmid overproducing wild type Pol IV (pDB10) or Pol IV^CD^ (pDB12) failed to transform in the presence of arabinose (Fig 2). However, in contrast to the other mutants, which formed tiny to small colonies in the presence of arabinose (Fig 2), the strain overproducing Pol IV^CD^-T120P displayed robust colonies that were indistinguishable from the strain bearing either the empty pBAD plasmid or overproducing Pol IV^LF^ (pDB14). In contrast to the robust growth observed for the strain overproducing Pol IV^CD^-T120P, the strain overproducing the full length Pol IV-T120P (pDB33) failed to grow in the presence of arabinose (Fig 2). We confirmed that the Pol IV^CD^-T120P mutant was expressed in a soluble form and at a level comparable to wild type Pol IV^CD^ (see legend to Fig 2). Based on this observation and results discussed later in this report, the difference between the growth phenotype of the strain expressing Pol IV^CD^-T120P and that expressing full length Pol IV-T120P appears to be a result of the Pol IV^LF^ domain, which contributes to the ability of Pol IV to impede *E*. *coli* growth [16,45]. Taken together, these findings suggest that a common mechanism underlies the ability of overproduced levels of Pol IV^CD^ to impede growth of the *dnaN* ^*+*^ strain and near-physiological levels of Pol IV to impede growth of the *dnaN159* strain. Furthermore, the fact that Pol IV^CD^ lacks the β clamp-binding Pol IV^LF^ domain, yet is nevertheless able to displace Pol III from the β clamp *in vitro* [16,45], suggests that Pol IV^CD^ interacts physically with one or more subunit of Pol III holoenzyme. Thus, the ability of T120P to alleviate the lethal phenotype may be indicative of this mutant being impaired for a Pol III-Pol IV interaction.

### Pol IV-T120P catalyzes DNA replication similar to wild type Pol IV *in vitro*

**Fig 3:**
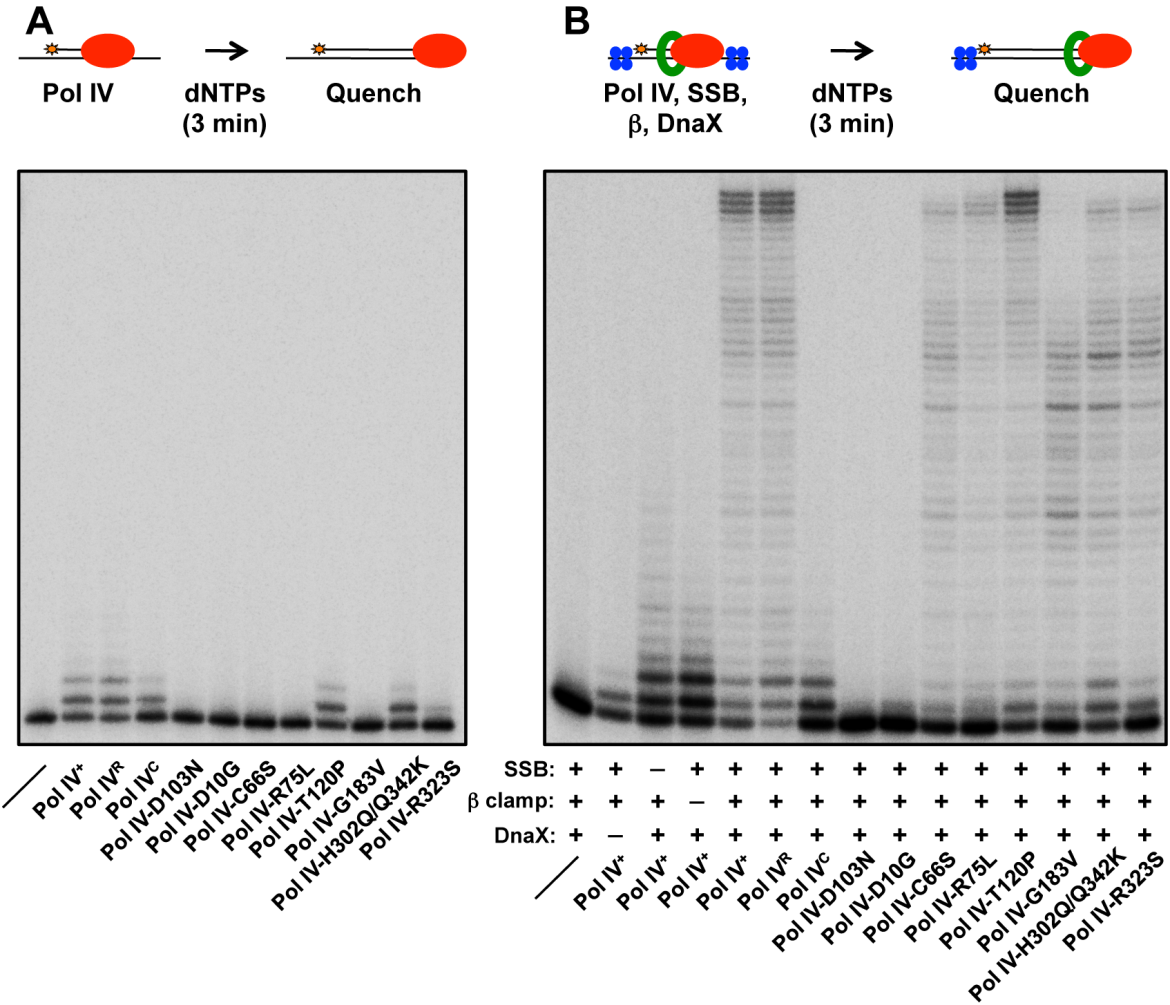
Ability of mutant Pol IV proteins to catalyze replication *in vitro*. Replication activity of the indicated Pol IV proteins (red) was measured **(A)** alone or **(B)** in the presence of the accessory proteins SSB (blue), β clamp (green) and DnaX using a primer extension assay as described in *Materials and Methods*.

In order to gain insight into the mechanistic basis for the phenotypes of the mutant Pol IV proteins, recombinant forms of each were purified for biochemical analyses. As noted above, we were able to overproduce all 13 mutant proteins. However, 6 of the 13 were either partially proteolyzed or poorly soluble following their overproduction from the T7 promoter (see Table 2), suggesting that their substitutions may affect the tertiary structure of Pol IV. The ability of the other 7 mutant Pol IV proteins to catalyze DNA replication *in vitro* was analyzed using a primer extension assay [17,34,38,56]. The DNA template consisted of a ^32^P labeled 30-mer annealed near the middle of a 100-mer (see depiction in Fig 3). Using this template, replication activity of each mutant Pol IV alone, as well as in the presence of single stranded DNA binding protein (SSB), β clamp and the DnaX (γ_3_δδ’) clamp loader accessory proteins was analyzed. As controls, we examined Pol IV-D103N, which lacks catalytic activity [17,38,57], as well as Pol IV^R^ and Pol IV^C^, which are impaired for interaction with the β clamp rim or cleft, respectively [38]. Based on published *in vitro* studies, the Pol IV-β clamp rim interaction is required for Pol III-Pol IV switching, but is dispensable for Pol IV replication. In contrast, the β clamp cleft interaction is required for both Pol IV replication and the Pol III-Pol IV switch [17].

In the absence of accessory proteins, Pol IV^R^, Pol IV^C^, Pol IV-T120P and Pol IV-H302Q/Q342K exhibited replication activity roughly comparable to wild type Pol IV (Fig 3A). In contrast, Pol IV-R323S was only modestly active, while Pol IV-D10G, Pol IV-C66S, Pol IV-R75L and Pol IV-G183V lacked detectable activity, similar to Pol IV-D103N (Fig 3A). In the presence of SSB, β clamp and the DnaX complex, Pol IV^R^ and Pol IV-T120P were again indistinguishable from wild type Pol IV (Fig 3B). Pol IV-C66S, Pol IV-R75L, Pol IV-G183V and Pol IV-R323S each displayed modest replication activity (Fig 3B), suggesting that the presence of accessory factors compensated in part for their intrinsic biochemical defects, possibly by helping to recruit the mutant Pol IV proteins to the primer/template junction, and/or by stabilizing an active conformation of the mutant Pol IV protein. Whereas Pol IV-H302Q/Q342K was indistinguishable from wild type Pol IV in the absence of accessory proteins (Fig 3A), it was impaired for processive replication in their presence compared to wild type (Fig 3B), suggesting that the Q342K mutation, which is adjacent to the Pol IV CBM (see Fig 1), interferes with the Pol IV-β clamp cleft interaction. Finally, Pol IV-D10G lacked detectable activity, similar to the D103N mutation, suggesting that D10 either participates directly in catalysis, or its substitution with glycine perturbs the structure of the catalytic center (see Fig 1D). Taken together, these results demonstrate that with the exception of Pol IV-D10G, each of the mutant proteins retained at least partial catalytic activity *in vitro*. Remarkably, Pol IV-T120P supported replication activity and processivity that were each comparable to that of wild type Pol IV.

In order to quantify the replication activity of Pol IV-T120P more rigorously, we measured its kinetic parameters and compared them to those of wild type Pol IV. As summarized in Table 3, the catalytic efficiency (k_pol_/K_d_) of Pol IV-T120P was ~2.5-fold higher than wild type Pol IV for incorporation of dC opposite template dG, and ~0.5-fold lower than wild type Pol IV for incorporation of dT opposite template dA. Both Pol IV and Pol IV-T120P were able to incorporate the other three dNTPs opposite a template dG or dA. However, in all cases the efficiency of misincorporation was significantly less (<10%) than that measured for correct incorporation. The small differences in catalytic efficiency between wild type Pol IV and Pol IV-T120P were attributable to effects of the T120P substitution on both dNTP binding (K_d_) and Pol turnover (k_pol_) (Table 3). Thus, despite the fact that residue T120 is well removed from the catalytic center of Pol IV (Fig 1), its substitution with a proline nevertheless exerts a modest effect on Pol IV catalysis. These findings, taken together with those discussed above, confirm that Pol IV-T120P retains essentially wild type Pol activity when replicating undamaged DNA, despite its inability to impede *E*. *coli* growth when expressed at elevated levels.

**Table 3:** Kinetic constants for wild type Pol IV and Pol IV-T120P nucleotide incorporation.

			Pol IV			Pol IV-T120P		
			K_d_	k_pol_	K_pol/_K_d_	K_d_	k_pol_	K_pol/_K_d_
DNA template ^*a*^	Template lesion	dNTP	(μM)	(s^-1^)	(M^-1^s^-1^)	(μM)	(s^-1^)	(M^-1^s^-1^)
13/20G	None	dCTP	>300	~5	~16,700	14.0	0.58	41,400
						±3.3	±0.04	±6,500
13/20MeG	*O* ^*6*^-mdG	dCTP	83	0.0060	72	60	0.0098	163
			±16	±0.0003	±18	±27	±0.0010	±40
13/20MeG	*O* ^*6*^-mdG	dTTP	121	0.0047	38	118.5	0.014	120
			±35	±0.0004	±15	±45.8	±0.002	±55
13/20A	None	dTTP	207	0.902	4,360	39	0.481	2,300
			±21	±0.040	±720	±9	±0.037	±2,100
13/20MeA	3d-medA	dTTP	350	0.345	990	34	0.050	1,470
			±100	±0.054	±200	±14	±0.007	±320
13/20Sp	AP	dATP	*ND* ^*b*^	*ND*	*ND*	460	0.0073	16
						±230	±0.0013	±8

^***a***^ The complete nucleotide sequence of each oligonucleotide is provided in S3 Table.

^***b***^ *ND*, not determined; accurate kinetic parameters could not be measured due to poor incorporation efficiencies at the highest dNTP concentration tested (500 μM).

### Mutant Pol IV proteins are impaired for TLS *in vivo*

**Fig 4:**
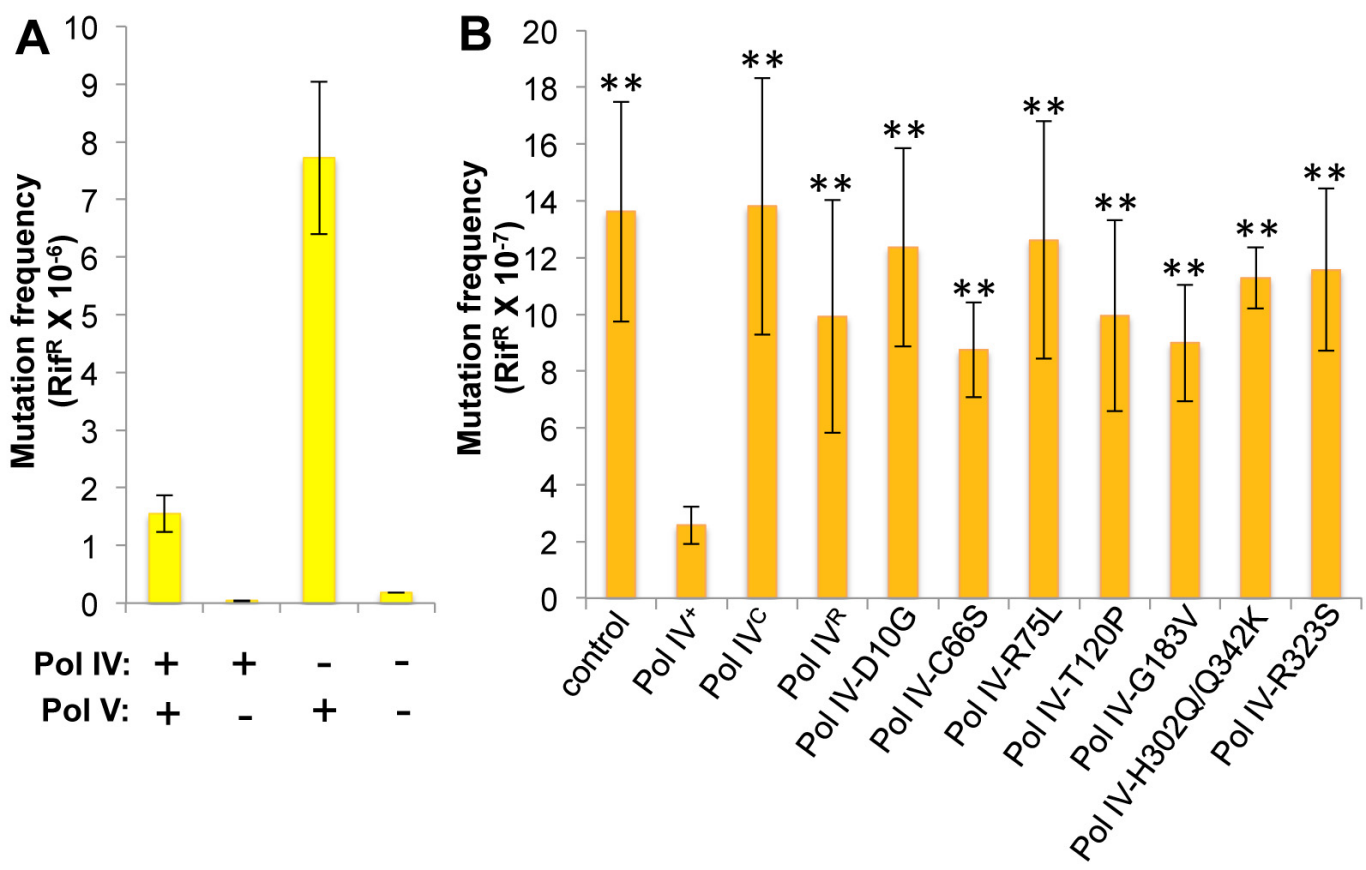
Ability of Pol IV mutants to tolerate MMS-induced DNA damage *in vivo*. **(A)** The dependence of MMS-induced mutagenesis on Pol IV (*dinB*) and Pol V (*umuDC*) function using strains RW118, RW120 (Δ*umuDC*), VB102 (Δ*dinB*), and VB103 (Δ*dinB* Δ*umuDC*), and **(B)** the ability of ~4-fold higher than SOS-induced levels of wild type or mutant Pol IV proteins (expressed from a plasmid) to suppress the frequency of Pol V-dependent MMS-induced mutagenesis of strain RW118 by competing with Pol V was measured as described in *Materials and Methods* and [33]. Results represent the average of at least 4 independent experiments ± one standard deviation. Symbols are as follows: **, *p*<0.0001; *, *p*<0.05.

Whereas Pol IV plays an important role in tolerating MMS-induced DNA damage by accurately bypassing lesions including *N* ^*3*^-mdA, Pol V (*umuDC*) contributes to MMS-induced mutations by mediating error-prone bypass of apurinic/apyrimidinic (AP) sites generated by either DNA glycosylases involved in the repair of alkylated bases, or their spontaneous decay [41,58]. Consistent with one published result [41], loss of Pol IV function resulted in a ~5-fold increase in the frequency of Pol V-dependent MMS-induced mutagenesis (Fig 4A). In contrast, expression of Pol IV at ~4-fold higher than SOS-induced levels from a low copy number plasmid (pRM102) reduced the frequency of MMS-induced mutagenesis ~5-fold compared to the pWSK29 empty vector control (Fig 4B). Taken together, we interpret these results to mean that Pol IV is limiting for accurate bypass of MMS-induced DNA lesions *in vivo*, and that when Pol IV is present, it leads to a reduction in the number of AP sites encountered by the replisome, thereby minimizing the Pol V-dependent mutator phenotype. In light of these findings, we asked whether any of the Pol IV mutants were able to reduce MMS-induced mutagenesis when expressed at ~4-fold higher than SOS-induced levels. In addition to the Pol IV mutants identified in the screen, we also analyzed Pol IV^R^ and Pol IV^C^. As summarized in Fig 4B, overexpression of Pol IV^R^ or Pol IV^C^ failed to reduce the frequency of MMS-induced mutagenesis (*p*<0.0001 based on Student’s *t*-test). Importantly, these results demonstrate a biologically important role for the β clamp rim and cleft in supporting Pol IV TLS function. Like Pol IV^R^ and Pol IV^C^, each of the other Pol IV mutants were also unable to reduce the frequency of MMS-induced mutagenesis compared to the strain expressing wild type Pol IV from pRM102 (*p*<0.0001 based on Student’s *t*-test). Taken together, these findings suggest that these mutant Pol IV proteins are impaired for gaining access to MMS-induced DNA adducts *in vivo* and/or mediating their bypass following recruitment, effectively shifting the bypass burden to Pol V.

### Pol IV-T120P is impaired for tolerating MMS-induced DNA damage *in vivo*

**Fig 5:**
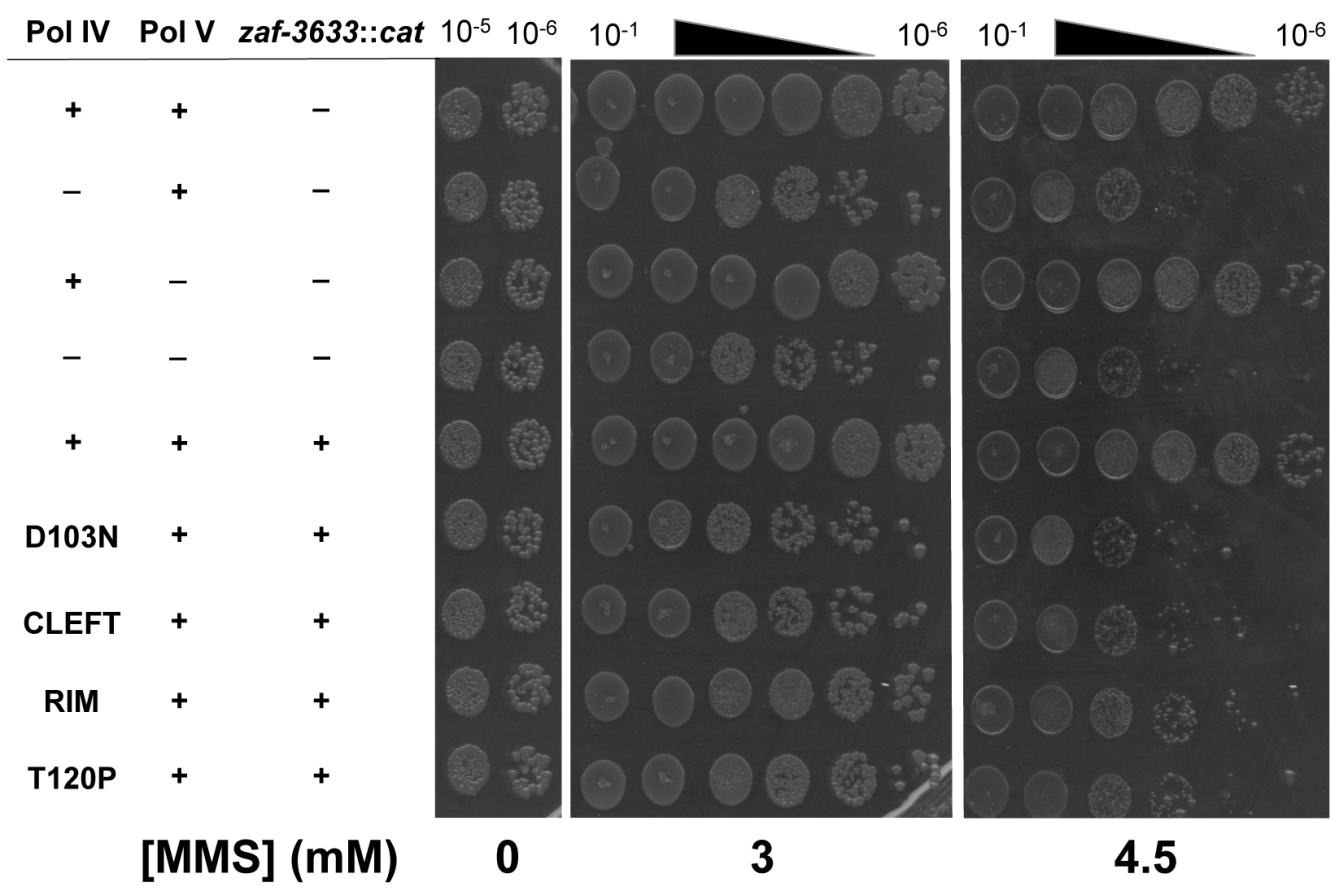
The Pol IV-T120P, Pol IV^C^ and Pol IV^R^ strains are sensitized to killing by MMS. Cultures of isogenic strains expressing the indicated Pol IV protein from the native *dinB* locus within the chromosome were serially 10-fold diluted using 0.8% saline, and 10 μl aliquots were spotted onto LB agar plates supplemented with the indicated concentrations of MMS. Results shown are representative of 4 independent experiments. The *zaf-3633*::*cat* allele, which is linked to the *dinB* locus and was used in strain construction, does not affect MMS sensitivity of the strains.

Since the Pol IV-T120P mutant retained complete catalytic activity while replicating undamaged DNA *in vitro* (Fig 3 and Table 3), but failed to accurately tolerate MMS-induced lesions *in vivo* when expressed at an elevated level (Fig 4), we more rigorously analyzed the TLS activity of Pol IV-T120P *in vivo* under physiologically relevant conditions. To this end, the *dinB* ^*+*^ allele was replaced with *dinB89*, which encodes the Pol IV-T120P mutation (see Table 2), and the ability of the resulting strain to tolerate MMS-induced DNA damage was measured. As controls, strains lacking *dinB* (Pol IV) and/or *umuDC* (Pol V), as well as Pol IV-D103N (*dinB80*), which lacks detectable Pol activity were used. In addition, we also constructed and analyzed Pol IV^R^ (*dinB82*) and Pol IV^C^ (*dinB81*) strains to gain insight into the biological importance of these contacts to Pol IV TLS. We first examined MMS sensitivity by spotting serial dilutions of respective cultures onto plates supplemented with 0, 3 or 4.5 mM MMS. Each of the Pol IV mutants displayed modest sensitivity to 3 mM MMS, with the Δ*dinB*, Pol IV-D103N, and Pol IV^C^ strains being slightly more sensitive than the Pol IV^R^ and Pol IV-T120P strains (Fig 5). At 4.5 mM MMS, the Δ*dinB*, Pol IV-D103N, and Pol IV^C^ strains were between ~100- to 1,000-fold more sensitive than the wild type Pol IV strain. The Pol IV^R^ and Pol IV-T120P strains were similar to each other, and were only slightly less sensitive than the Δ*dinB* strain (Fig 5). Taken together, these results demonstrate a biologically important role in Pol IV TLS for the β clamp rim as well as residue T120 of Pol IV. In contrast to Δ*dinB*, the Δ*umuDC* strain failed to display MMS sensitivity, or to exacerbate sensitivity of the Δ*dinB* strain, consistent with published reports [41]. Finally, MMS sensitivity of each of the Pol IV mutant strains was fully complemented by pRM102, which expresses wild type Pol IV (S5A Fig).

**Fig 6:**
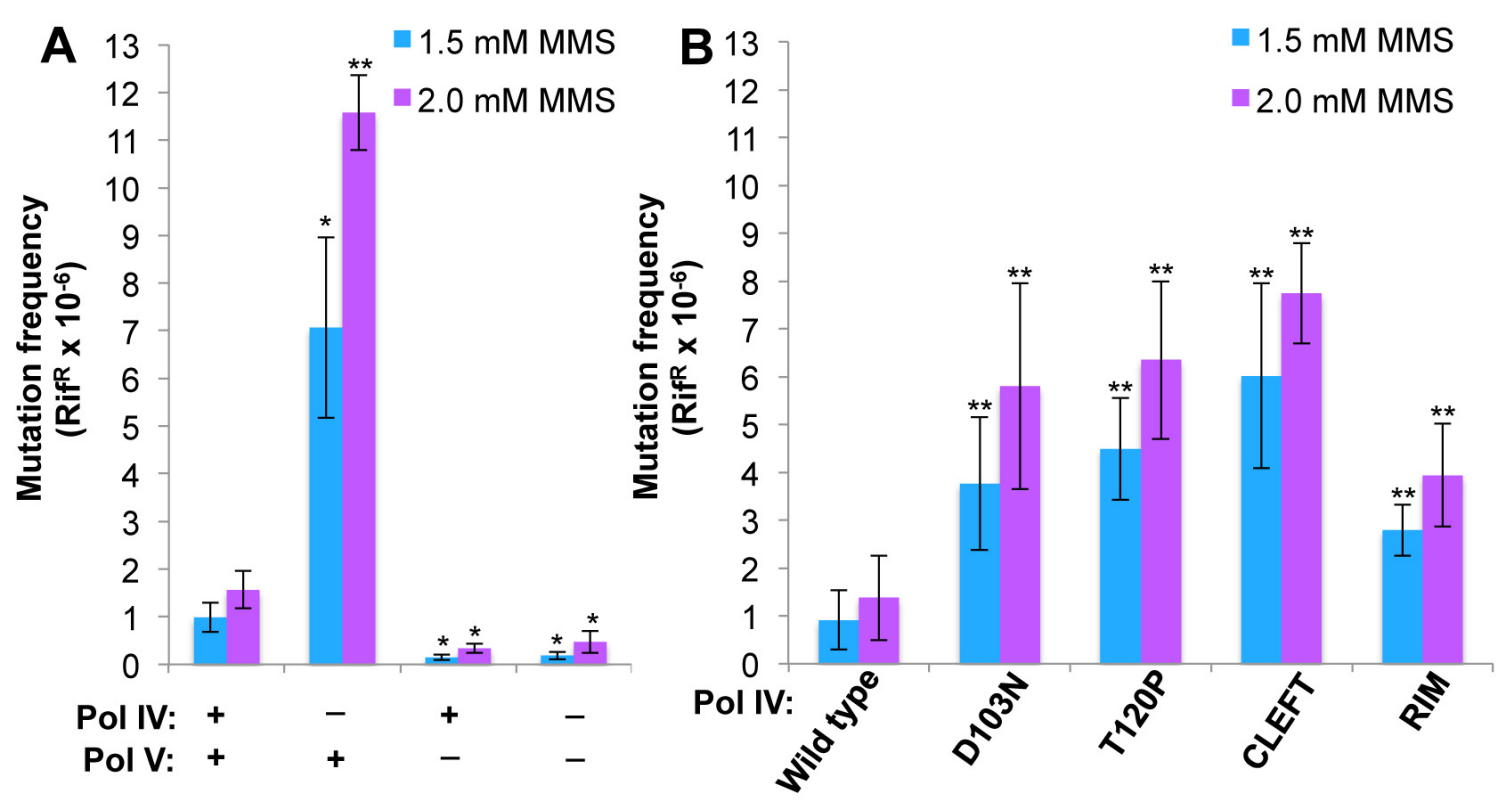
The Pol IV-T120P, Pol IV^C^ and Pol IV^R^ strains display an increased frequency of MMS-induced mutagenesis. **(A)** The dependence of MMS-induced mutagenesis on Pol IV (*dinB*) and Pol V (*umuDC*) function using strains MG1655, MKS101 (Δ*umuDC*), MKS100 (Δ*dinB*), and MKS102 (Δ*dinB* Δ*umuDC*). **(B)** The ability of wild type or mutant Pol IV proteins to suppress the frequency of Pol V-dependent MMS-induced mutagenesis when expressed from the chromosome was measured as described in *Materials and Methods* [33]. Results represent the average of at least 4 independent experiments ± one standard deviation. Symbols are as follows: **, *p*<0.0001; *, *p*<0.05.

We next measured the frequency of MMS-induced mutagenesis. The strain lacking Pol IV displayed a ~7-fold increase in Pol V-dependent MMS-induced mutagenesis (Fig 6A), as expected [41]. Frequencies for the Pol IV-D103N (*dinB80*), Pol IV-T120P (*dinB89*), Pol IV^C^ (*dinB81*) and Pol IV^R^ (*dinB82*) strains were ~4-, ~5-, ~6- and ~3-fold elevated, respectively, relative to the wild type Pol IV control (Fig 6B; *p*≤0.0001 based on Student’s *t*-test). These results demonstrate the importance of position T120 in Pol IV, as well as the ability of Pol IV to interact with the β clamp rim and cleft to carry out TLS *in vivo*. Importantly, the ability of Pol IV to inhibit mutagenesis by Pol V was not affected by the presence of the *zaf-3633*::*cat* cassette (*p* = 0.7 based on Student’s *t*-test comparing the Pol IV^+^ strains MG1655 and MKS103). As with MMS sensitivity, wild type Pol IV expressed from plasmid pRM102 restored the frequency of MMS-induced mutagenesis for these *dinB* mutants to the wild type Pol IV level (S5B Fig).

To determine if the MMS phenotypes of the Pol IV-T120P strain were due to a catalytic TLS defect which rendered Pol IV-T120P incapable of bypassing MMS-induced lesions, we used a primer extension assay to measure the ability of purified Pol IV-T120P to catalyze *in vitro* bypass of the model MMS-induced lesions *O* ^*6*^-methylguanine (*O* ^*6*^-mdG), 3-deaza-3-methyladenine (3d-medA), which is a stable mimic of *N* ^*3*^-mdA [59], as well as an AP site. As summarized in Table 3, both wild type Pol IV and Pol IV-T120P were able to bypass template *O* ^*6*^-mdG and 3d-medA. While there were some differences in substrate binding (K_d_) and/or turnover (k_pol_), Pol IV-T120P was as efficient or more so than wild type Pol IV. Pol IV and Pol IV-T120P each incorporated either dC or dT opposite template *O* ^*6*^-mdG with roughly equivalent efficiencies. In both cases, bypass was considerably less efficient than that observed for template dG, due to a reduction in both K_d_ and k_pol_, with Pol IV-T120P slightly outperforming wild type Pol IV. Both Pols were capable of incorporating low levels of dA or dG opposite template *O* ^*6*^-mdG. However, the amount of incorporation was less than 10% compared to that for the insertion of dC or dT opposite the alkylated lesion. 3d-medA was easier for both Pol IV and Pol IV-T120P to bypass, again with Pol IV-T120 outperforming wild type Pol IV by a factor of ~2-fold, attributable in large part to stronger substrate binding (K_d_). Both Pols incorporated dA, dC or dG opposite template 3d-medA. The level of incorporation was significantly less than that measured for incorporation of dT. Finally, even though Pol IV-T120P was marginal in regard to its ability to bypass the AP site, inserting dA, it was nevertheless more efficient than wild type Pol IV (Table 3). Taken together, these results demonstrate that Pol IV-T120P is proficient *in vitro* for TLS past a variety of DNA adducts commonly induced by MMS, suggesting that the inability of Pol IV-T120P to cope with MMS-induced DNA damage *in vivo* was the result of its inability to gain access to the lesions.

### Pol IV^CD^-T120P fails to displace an actively replicating Pol III from β clamp *in vitro*

**Fig 7:**
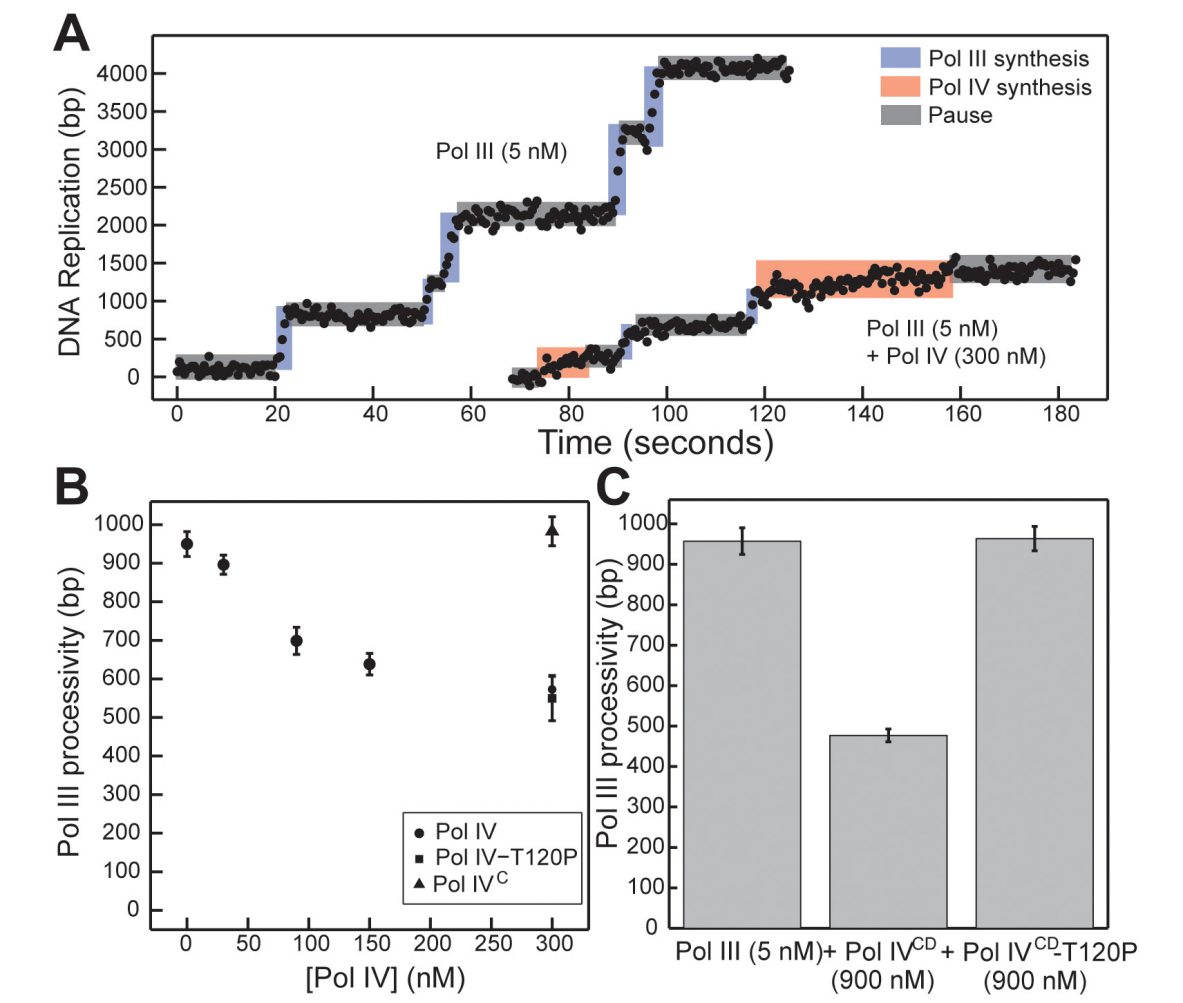
Pol IV^CD^-T120P, but not full length Pol IV-T120P, fails to inhibit Pol III processivity *in vitro*. **(A)** Representative trajectories for primer extension of individual DNA molecules by Pol III alone (5 nM), or in the presence of excess Pol IV (300 nM). Processive events are marked in blue (Pol III) or red (Pol IV), with intervening pauses in grey. **(B)** A reduction in Pol III processivity in the presence of excess Pol IV (circles) can be abrogated by removing the Pol IV CBM (Pol IV^C^, triangle), but not by the T120P mutation (square, note overlap). Each point represents the mean of 50–470 processive events ± the standard error of the mean. **(C)** The catalytic domain of Pol IV (Pol IV^CD^, 900 nM) disrupts active Pol III (5 nM) synthesis, while an equivalent concentration of Pol IV^CD^-T120P does not. Bars represent the mean of 470, 301 and 657 separate processive events, respectively, ± the standard error of the mean.

We previously utilized a single molecule primer extension assay to demonstrate exchange of Pol III and Pol IV on β clamp at the 3’ primer terminus *in vitro* [12]. The distinct polymerization rates of Pol III and Pol IV allowed us to unambiguously assign individual DNA synthesis events to each respective Pol and to measure their respective processivities when incubated alone or together (Fig 7A). At 300 nM Pol IV, a 60-fold molar excess over Pol III that simulates levels found in SOS-induced cells [3], Pol IV actively displaced Pol III from the DNA template as inferred from Pol III processivity measurements ([12,16,17,38,45]; see Fig 7B). This ability of Pol IV to reduce Pol III processivity was dependent on the Pol IV CBM, arguing that Pol III displacement involves at a minimum a conformational exchange of the two Pols on the β clamp. Using this approach, we asked whether Pol IV-T120P was likewise able to displace Pol III from the β clamp, as inferred by a reduction in its processivity. As summarized in Fig 7B, a 60-fold molar excess of Pol IV-T120P over Pol III was as efficient as wild type Pol IV at inhibiting Pol III processivity. Together, these results suggest one of two possibilities: either (i) the T120P mutation does not impact the ability of Pol IV to displace Pol III from β clamp, or (ii) efficient recruitment of Pol IV to the Pol III-β clamp complex through its interactions with β clamp masks the Pol IV-T120P-dependent defect in Pol IV displacement of Pol III from β clamp. To distinguish between these two models, we analyzed the ability of Pol IV^CD^ and Pol IV^CD^-T120P, both of which lack the Pol IV^LF^ β clamp-binding domain, to impede Pol III processivity using the same single molecule assay. Furukohri and colleagues previously demonstrated that a ~900- to 1,800-fold molar excess of Pol IV^CD^ over Pol III (890 nM Pol IV compared to 0.5–1.0 nM Pol III) was able to disrupt the Pol III-β clamp complex assembled *in vitro* on a primed DNA substrate [16]. Similarly, we found that 900 nM Pol IV^CD^ (a 180-fold molar excess over Pol III) disrupted Pol III synthesis, reducing its processivity to one-half of that observed in the absence of Pol IV^CD^ (Fig 7C, *p <*0.01, determined using the Wilcoxon rank-sum test). This reduction in Pol III processivity most likely results from the ability of Pol IV^CD^ to displace Pol III from the β clamp assembled on DNA [12,16,45]. Importantly, an equivalent concentration of Pol IV^CD^-T120P failed to reduce processivity of Pol III. Taken together, these findings support the view that residue T120 of Pol IV plays an important role in displacing Pol III from the β clamp, and demonstrate that the Pol IV^LF^ domain contributes to this ability. Finally, these biochemical results are remarkably similar to the growth phenotypes observed for strains overproducing Pol IV^CD^-T120P (pDB23) or full length Pol IV-T120P (pDB33) from the arabinose promoter (Fig 2), which demonstrate the ability of the Pol IV^LF^ domain to mask the phenotype of the T120P mutation *in vivo*.

## Discussion

With the goal of gaining new insights into the relationship between the physiological function of Pol IV in TLS and its ability when overexpressed to impede *E*. *coli* growth, we exploited the hypersensitivity of the *dnaN159* strain to elevated levels of Pol IV to identify 13 novel Pol IV mutants that were unable to impede growth (Table 2). These Pol IV mutants were deficient in stimulating reversion of the CC108 *lacZ* –1 frameshift reporter when expressed at SOS-induced levels (S2 Fig), and for conferring UV sensitivity upon the *dnaN159* strain (S3 Fig), indicating that they were unable to effectively compete with Pol III for access to the replication fork. Likewise, these mutations failed to impede growth of the *dnaN* ^*+*^ strain when introduced into Pol IV^CD^ and overproduced from the arabinose promoter (Fig 2). Finally, despite the fact that all but one of the mutant Pol IV proteins (Pol IV-D10G) retained appreciable catalytic activity *in vitro* (Fig 3), they were nevertheless impaired for tolerating MMS-induced lesions *in vivo* (Fig 4). These results, taken together with those discussed below, support the view that overexpressed levels of Pol IV impede *E*. *coli* growth by actively replacing Pol III at the replication fork *via* a mechanism that is similar to that used under physiological conditions to coordinate high fidelity processive Pol III replication with potentially mutagenic Pol IV TLS. In contrast to an earlier study [47], we failed to observe an ability of overproduced levels of Pol IV to mediate cell death in either the *dnaN159* or *dnaN* ^*+*^ strains *via* excessive incorporation of oxidized precursors (S2 Table and S4 Fig). Thus, Pol IV appears to be able to impede *E*. *coli* growth by either incorporating lethal levels of 8-oxo-dG or by displacing Pol III. This view is consistent with the finding that under the conditions used in this study lethality was independent of Pol IV catalytic activity [38,45]. However, our finding that several Pol IV mutants identified in this work were impaired for catalytic activity *in vitro* (Fig 3) suggests that the ability of Pol IV to replace Pol III at the replication fork is dependent at least in part on residues in Pol IV that contribute to catalytic activity. Alternatively, the ability of Pol IV to persist at the replication fork after replacing Pol III likely contributes to the killing, and would rely on Pol IV processivity, which, with the exception of Pol IV-T120P, was impaired in the Pol IV mutants analyzed here.

The Pol IV-T120P mutant was remarkable in that it was comparable to wild type Pol IV for replication of undamaged DNA *in vitro*, as well as for catalyzing bypass of 3d-medA, *O* ^*6*^-mdG and an AP site (Fig 3 and Table 3), yet it was nevertheless unable to tolerate MMS-induced DNA lesions *in vivo* (Figs 4–6), presumably due to its inability to access these lesions. Consistent with this conclusion, the T120P mutation abrogated the ability of Pol IV^CD^ to inhibit Pol III processivity *in vitro* (Fig 7). Based on previously published results [12,16,45], inhibition of Pol III processivity is the result of Pol IV displacing Pol III from the face of the β clamp. Our finding that the Pol IV-T120P strain was impaired for tolerating MMS-induced DNA damage indicates that the ability of Pol IV to inhibit Pol III processivity is required for the TLS function of Pol IV *in vivo*. Furthermore, our finding that the strain expressing Pol IV-T120P (*dinB89*) was almost as deficient as the isogenic Δ*dinB*, Pol IV-D103N (*dinB80*) and Pol IV^C^ (*dinB81*) strains for tolerating MMS-induced lesions indicates that the ability of Pol IV to displace Pol III from the face of the β clamp is critical to Pol IV TLS function *in vivo* (Figs 5 and 6). TLS has been suggested to take place at either the replication fork *via* a Pol switch [3,12,15,17,18,20], or in ssDNA gaps generated in part by Pol III skipping over DNA lesions to continue replication downstream of the blockage [18,39]. However, to date, the extent to which these two mechanisms are used *in vivo* was unknown. Our finding that the T120P mutation specifically interferes with the ability of Pol IV to switch with Pol III, taken together with its inability to cope with MMS-induced DNA damage *in vivo* (Figs 4–6), suggests that a significant fraction of Pol IV-mediated TLS *in vivo* relies on a Pol III-Pol IV switch. Consistent with this conclusion, SOS-induced levels of Pol IV slowed the rate of DNA replication *in vivo* by ~12%, arguing that Pol IV frequently replaces Pol III at the replication fork following SOS induction [14], possibly *via* a Pol III-Pol IV switch. While it remains to be determined whether the reduced rate of replication in response to SOS induction represents a biologically important checkpoint effector function of Pol IV, as previously suggested [13,45], the fact that strains lacking Pol IV function fail to exhibit enhanced sensitivity to agents that generate classes of DNA lesions other than those that Pol IV is capable of bypassing, such as UV photoproducts [60], argues against such a model. Finally, Benson and colleagues [61] identified two Pol IV mutants (V7G and F292Y) based on their inability to impede growth of an *E*. *coli* strain expressing a mutant Pol IIIα allele (*dnaE915*). Both of these mutants retained the ability to bypass 3d-medA *in vitro*, but their abilities to cope with MMS-induced DNA damage *in vivo* was not examined. While V7 is in close proximity to T120 (S6A Fig), neither it nor F292 is surface exposed (S6B Fig), suggesting that these mutations may affect the structure of Pol IV. Regardless, it is possible that the V7G and/or F292Y mutations impair the Pol III-Pol IV switch.

We previously described results supporting an important role for the Pol IV-β clamp rim interaction in mediating the Pol III-Pol IV switch *in vitro* [17,38]. In contrast to our findings, Gabbai and colleagues, utilizing a different assay that may more accurately represent the structure and composition of the replisome, concluded that the Pol IV-β clamp rim contact stimulated, but was not required for a Pol III-Pol IV switch *in vitro* [18]. In light of this finding, they suggested that direct competition between Pol III and Pol IV for the β clamp cleft represented an alternative mechanism for their switching. Previous efforts to define the role of the β clamp rim in Pol IV function *in vivo* utilized multi-copy plasmids expressing higher than physiological levels of Pol IV^R^ and Pol IV^C^ to complement the NFZ-sensitive phenotype of a Δ*dinB* strain [17,62]. Under these conditions, the Pol IV^R^ strain was indistinguishable from the wild type Pol IV strain, suggesting the Pol IV-β clamp rim interaction was dispensable for Pol IV function *in vivo*. However, using strains expressing the Pol IV^C^ or Pol IV^R^ mutants from the chromosomal *dinB* locus, we confirmed an essential role for the β clamp cleft in Pol IV TLS, and provide compelling evidence for a biologically important role for the β clamp rim in contributing to Pol IV TLS (Figs 4–6). These findings, taken together with those discussed above regarding the T120P mutation, support the model that Pol IV TLS function *in vivo* relies on its ability to bind to both the rim and cleft of the β clamp, as well as its ability to inhibit Pol III processivity, possibly *via* a direct interaction of Pol IV with one or more subunits of Pol III. Since both the Pol IV-β clamp and the postulated Pol IV-Pol III interactions are required for Pol IV TLS function *in vivo*, it is conceivable that the postulated Pol III-Pol IV interaction could act to relax the requirement for the Pol IV-β clamp rim interaction *in vitro*, potentially explaining the apparent discrepancy between our published results and those of Gabbai and colleagues regarding the importance of the β clamp rim to the Pol III-Pol IV switch *in vitro*.

Pol IV interacts physically with both UmuD and RecA [63–65]. These interactions are proposed to improve the fidelity of Pol IV by enclosing its open active site [64]. Interestingly, Pol IV-C66A was previously reported to bind more tightly to both RecA and UmuD [65]. It is conceivable that we identified the Pol IV-C66S with our genetic assay because it was affected for interactions with UmuD and/or RecA. Alternatively, we may have identified Pol IV-C66S due to its reduced stability ([65]; see S1 Fig). In addition to C66, residues P166, F172 and L176 of Pol IV have also been demonstrated to interact with UmuD [64]. In contrast to C66, these residues are in close proximity to T120 (S6 Fig, panels C and D). Thus, it is possible that the T120P mutation also affects an interaction of Pol IV with UmuD. Finally, UmuD may contribute to the ability of Pol IV to switch with Pol III. Consistent with this possibility, UmuD interacts physically with the Pol IIIα and Pol IIIε subunits, as well as the β clamp [21,22,66]. However, the failure of Pol IV^CD^-T120P to impede Pol III processivity *in vitro* was independent of UmuD (Fig 7C).

**Fig 8:**
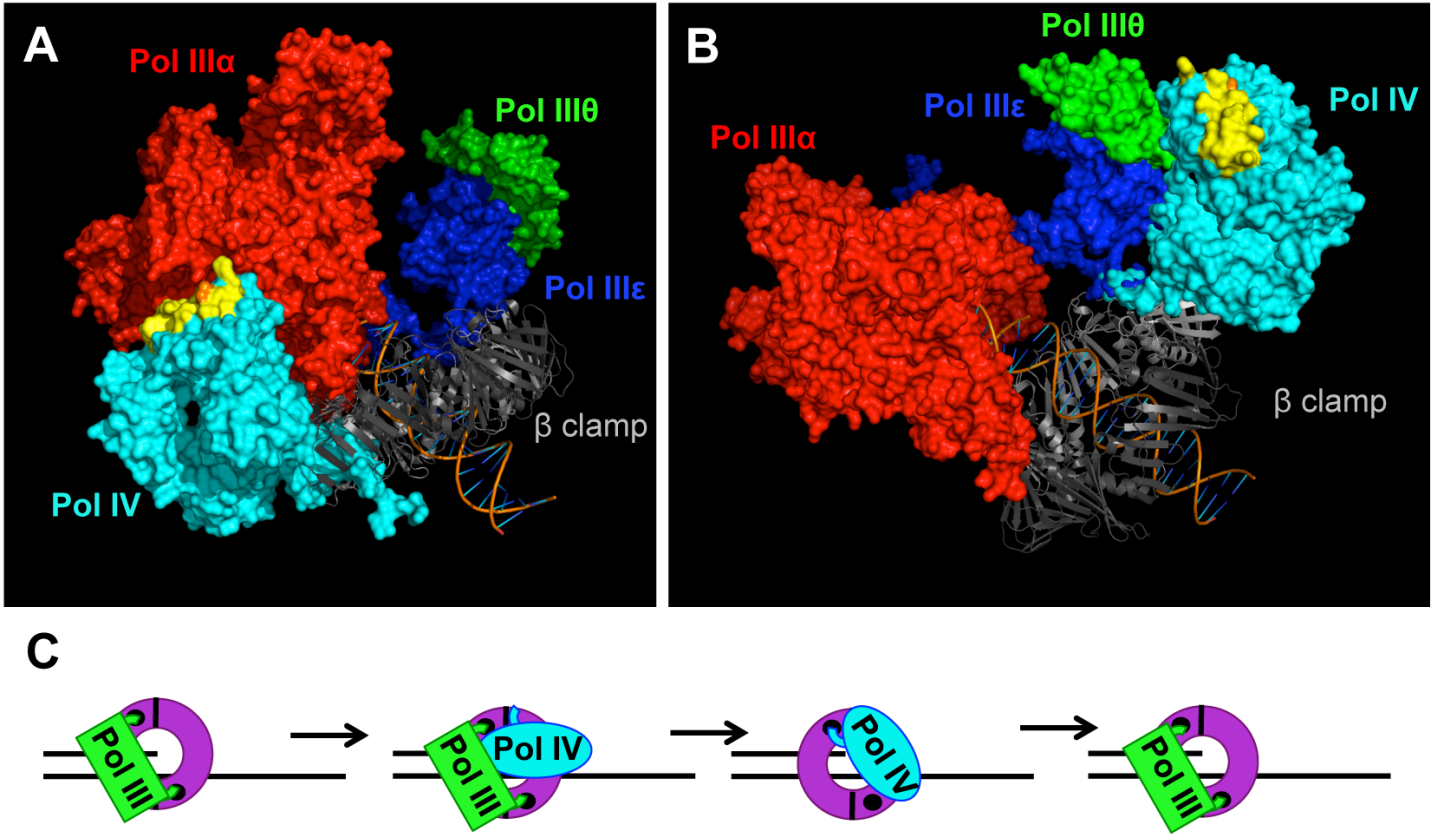
Models for the role of residue T120 in Pol IV function. Models for the structure of the Pol IIIαεθ-β clamp-DNA complex with Pol IV bound to the rim of the β clamp that is immediately adjacent to the β cleft that is bound by either **(A)** Pol IIIα (red) or **(B)** Pol IIIε (blue). These models were built using MacPyMol Molecular Graphics System, Version 1.7.4 Schrödinger, LLC and the previously published model for the Pol IIIαεθ-β clamp-DNA complex [84]. Pol IV (PDB 4IR9) was docked onto the rim of the β clamp in complex with Pol IIIαεθ by aligning it with the Pol IV^LF^ domain in PDB 1UNN [12,53]. Residue T120 of Pol IV is in orange, while α-helix 5 corresponding to residues H116-Q135 of Pol IV is in yellow. **(C)** Model of the Pol III-Pol IV switching mechanism in which Pol IV (cyan) gains access to the DNA template after making contact with one or more subunits of Pol III (green), as well as the rim of the β clamp (purple). The small black circles represent the clefts in the β clamp. See text for further details.

Results discussed in this report provide several new insights into the mechanism by which the actions of Pol III are coordinately regulated with those of Pol IV, and when taken together with previously published findings [17,33,38,67], support a new model for the role of Pol III-Pol IV switching in Pol IV-mediated TLS *in vivo*. In addition to its interactions with the β clamp, an interaction of Pol IV with Pol III also appears to play a biologically important role in recruiting Pol IV to lesions (Figs 5 and 6). Biochemical interaction of Pol IV^CD^ with Pol III holoenzyme is sufficient to mediate displacement of Pol III from the face of the β clamp *in vitro* [12,13,16,45]. Our single molecule assay reproduced this finding, and further demonstrated that position T120 of Pol IV is important for this function (Fig 7). Residue T120 is one helical turn from the start of α-helix 5 (see Fig 8) and its substitution with proline likely truncates the start of this helix. Thus, residue T120, and/or residues in its vicinity, presumably mediates a physical interaction with one or more subunits of the Pol III holoenzyme. Both the α catalytic and the ε proofreading subunits of Pol III contain a CBM that interacts with the β clamp cleft [68,69]. Although the Pol III holoenzyme binds both β clamp clefts, a single β clamp cleft is sufficient to support processive Pol III replication, as well as Pol III-Pol IV switching [17,36,67]. Since the Pol IIIα-β clamp cleft interaction is required for Pol III function both *in vitro* and *in vivo*, while the Pol IIIε-β clamp cleft appears to be dispensable [17,29,36,67,69], we suggest that a mechanism by which Pol IV initiates a switch with a stalled Pol III involves Pol IV first binding to the β clamp rim adjacent to the β clamp cleft that is bound by Pol IIIα. Based on an *in silico* model of the Pol III-β clamp-Pol IV complex, residue T120 of Pol IV is well positioned to contact Pol IIIα (Fig 8A), but not Pol IIIε (Fig 8B). Thus, Pol IV may be recruited to the replication fork through a combination of the Pol IV^LF^-β clamp rim and Pol IV^CD^-Pol III interactions. The Pol IV^LF^-β clamp rim interaction is likely too weak (~1.3 μM) on its own to recruit Pol IV to the replisome in the absence of SOS-induction when Pol IV levels are ~330 nM [3,70]. However, if a Pol IV-Pol III interaction contributes to Pol IV recruitment, the affinity of Pol IV for the replisome may be sufficiently high to enable a Pol III-Pol IV switch irrespective of SOS-induction, which increases Pol IV levels from ~330 nM to ~3.3 μM [3,70].

Pol IV can only switch with a stalled Pol III [15,38], suggesting that a conformational change in Pol IIIα contributes to the Pol III-Pol IV switch, possibly by unmasking a surface of Pol III that interacts with Pol IV leading to displacement of Pol III. It is currently unclear whether Pol IV is recruited to the replication fork in response to a specific conformation of the stalled Pol III replisome, or whether it is recruited through one or more Pol III-Pol IV interactions that are independent of a stalled Pol III. If a stalled Pol III replisome acts to recruit Pol IV, then a single Pol III-Pol IV interaction may be sufficient for both recruitment and Pol III displacement. However, if an actively replicating Pol III replisome recruits Pol IV irrespective of Pol III stalling, then distinct Pol III-Pol IV contacts would seemingly be required for Pol IV recruitment and Pol III-Pol IV switching. Finally, irrespective of the fact that the Pol IV-β clamp rim interaction is required for Pol IV-mediated TLS *in vivo* (Figs 5 and 6), the finding that Pol IV^CD^ displaced Pol III from the face of the β clamp ([16]; see Fig 7) suggests that Pol IV may not have to simultaneously bind the β clamp rim as well as one or more subunits of Pol III in order to displace Pol III from the face of the β clamp. Irrespective of the mechanism, once Pol III is displaced, the C-terminal 6 residues of Pol IV are able to bind the cleft of the β clamp that was previously bound by Pol III, ultimately granting control of both the β clamp and the replication fork to Pol IV for TLS (Fig 8C). Although Pol IV displaces Pol III from the β clamp [12,16,45], we suggest that Pol IIIα-DnaXτ-DnaB interactions act to retain Pol III within the replisome complex until such time as Pol IV relinquishes its control of the DNA template [71], allowing Pol III to regain control of the replication fork after Pol IV leaves. However, when Pol IV is overexpressed, or when the replisome contains the mutant *dnaN159*-encoded β clamp protein, Pol IV repeatedly replaces Pol III on the clamp face. This repeated replacement could act to displace Pol III from the replisome, explaining the lethal phenotype observed for strains overexpressing Pol IV. While the Pol IV-β clamp rim interaction is presumably dispensable once Pol IV gains access to the β clamp cleft [17], processive Pol IV replication requires contact with residues ^148^HQDVR^152^ of β clamp [33]. Inasmuch as residues H148, Q149 and R152 of β clamp interact with the DNA template that it encircles [33,72], Pol IV may have to compete with DNA to gain access to ^148^HQDVR^152^ of β clamp, which, in turn, may act to reposition the β clamp on the DNA, possibly exposing additional surfaces on the β clamp that stabilize the Pol IV-β clamp complex, and/or enhance its catalytic activity. Finally, our finding that the Pol IV^R^, Pol IV^C^ and Pol IV-T120P mutant strains were severely impaired for tolerating MMS-induced DNA damage is consistent with the view that Pol III-Pol IV switching plays a pivotal role in regulating access of Pol IV to the DNA *in vivo*. Further studies are required to determine how long Pol IV maintains control of the replication fork after switching with Pol III, as well as whether Pol III and/or other factors play a role in displacing Pol IV from the replication fork.

## Materials and Methods

### *E*. *coli* strains, plasmid DNAs and bacteriological techniques

Bacteria were cultured in either Luria Bertani (LB; 10 g/l tryptone, 5 g/l yeast extract, 10 g/l NaCl), or M9 minimal medium (12.9 g/L Na_2_HPO_4_•7H_2_O, 3 g/L KH_2_PO_4_, 0.5 g/L NaCl, 1 g/L NH_4_Cl) supplemented with 0.1 mM CaCl_2_, 2 mM MgCl_2_, 5 μg/ml thiamine, 0.5% casamino acids and 0.5% glucose or 0.2% arabinose, as indicated. For anaerobic growth, 100 mM KNO_3_ was added to the growth to act as the terminal electron acceptor. When required, the following antibiotics were used at the indicated concentrations: ampicillin (Amp), 150 μg/ml; tetracycline (Tet), 10 μg/ml; kanamycin (Kan), 40 μg/ml; chloramphenicol (Cam), 20 μg/ml; rifampicin (Rif), 50 μg/ml. *E*. *coli* strains were constructed using P1*vir*-mediated generalized transduction [73], or λRed-mediated recombineering [74], and are described in Table 1. Strain genotypes were verified using either diagnostic PCR or nucleotide sequence analysis (Roswell Park Biopolymer Facility, Buffalo, NY) of respective PCR-amplified alleles. Strains were made competent for transformation using CaCl_2_ as described [48]. Bacterial plasmid transformation frequency [38], UV sensitivity [38,48] and *lacZ*→Lac^+^ reversion [51,52,56] was measured as described in the indicated references. Plasmid DNAs are described in Table 1. Standard techniques were used for cloning. Site-directed mutagenesis was performed using the Quickchange kit (Stratagene). Synthetic oligonucleotide primers used for mutagenesis were purchased from either IDT or Operon, and their sequences are presented in S3 Table. All plasmid sequences were confirmed by nucleotide sequence (Roswell Park Biopolymer Facility, Buffalo, NY). Mutant *dinB* alleles were subcloned from pWSK29 into pET11a (Novagen) by NdeI and BamHI (Fermentas) restriction, followed by ligation to the similarly prepared pET11a backbone using T4 DNA ligase (Fermentas). Sensitivity to MMS (Sigma) was measured as described [41].

### Genetic assay to identify Pol IV mutants

Spontaneous *dinB* mutations unable to impede growth of the *dnaN159 lexA51*(Def) *E*. *coli* strain MS105 were identified by selecting Amp^R^ transformants using 200 ng of plasmid pJH110. This strain displayed a transformation efficiency of ~10^6^ colony forming units/μg of supercoiled pWSK29 plasmid DNA. In instances where multiple colonies were obtained from a single transformation reaction, a single CFU was selected from the plate for further analysis to avoid sibling mutations. Plasmids were isolated using the Qiagen mini-prep kit as per the manufacturer’s recommendation. Purified plasmids were analyzed by agarose gel electrophoresis. Those of the appropriate size were retransformed into MS105 to verify their inability to impede growth. Those that transformed MS105 with an efficiency similar to that of the pWSK29 control plasmid were then analyzed by Western blotting using polyclonal anti-Pol IV antibodies as described [4,17].

### Construction of mutant *dinB* strains

Strains MKS103-MKS107 were constructed using λ recombineering as described [74]. Briefly, the 2,329 bp *lafU’ zaf-3633*::*cat dinB80 yafN’* DNA cassette was PCR amplified from plasmid pMKS100, pMKS101, pMKS102, pMKS103 or pMKS104 using primers P1 and P4 (S3 Table), and electroporated into *E*. *coli* strain MG1655 containing pKD46 as described [75]. Chloramphenicol resistant colonies were selected on LB agar plates supplemented with chloramphenicol, and subsequently confirmed to contain the desired *dinB* allele by diagnostic PCR using primers MKS055 and MKS046, which anneal 589 bp upstream of primer P1 and 498 bp downstream of primer P4, respectively. The remaining primers listed in S3 Table were used for nucleotide sequence verification of the *lafU’–zaf-3633*::*cat–dinB*–*yafN’* cassette from 91 bp upstream of the start of primer P1 to 163 bp downstream from the end of primer P4, except for a 153 bp internal segment of the *cat* gene corresponding to amino acid residues L45-D96, prior to using P1*vir* to transduce the linked *zaf-3633*::*cat* and *dinB* alleles into a fresh isolate of strain MG1655.

### Measure of MMS-induced mutation frequency

Cultures of LB media (mock samples to measure spontaneous mutagenesis) and LB media containing the indicated concentration of freshly added MMS (1.5 mM or 2.0 mM) were inoculated with 200 μl of an exponential culture (OD_600_ ~0.5) of the indicated strain. Cultures were incubated overnight at 37°C with aeration before plating appropriate dilutions onto LB media with or without Rif. Plates were incubated overnight at 37°C before counting colonies. MMS-induced mutation frequency was calculated as described [33].

### *In vitro* DNA primer extension assay

Wild type and Pol IV mutant proteins [34], SSB [76], the γ_3_δδ’ form of the DnaX clamp loader [36] and β clamp [77] were purified as described in the indicated references. Primer extension assays were performed as described previously [17,34,56] using the ^32^P-radiolabeled PAGE purified 30-mer/100-mer DNA template. Briefly, reactions (20 μl) contained replication buffer (20 mM Tris-HCl [pH 7.5], 8.0 mM MgCl_2_, 0.1 mM EDTA, 5 mM DTT, 1 mM ATP, 5% glycerol, and 0.8 μg/ml BSA), 1 nM 30-mer/100-mer template, 133 μM dNTPs (Fermentas), 90 nM SSB, 1 nM γ_3_δδ’ DnaX clamp loader complex, 10 nM β clamp and 1 nM Pol IV. The reactions were pre-incubated for 3 min at 37°C to permit loading of β clamp prior to initiating replication by addition of dNTPs. Reactions were next incubated at 37°C for 5 min, then quenched by the addition of 25 mM EDTA and incubation at 95°C for 3 minutes. Aliquots of each reaction were then electrophoresed through an 8% UREA-PAGE at 60 watts for 3,332 volt hours, as described [56]. Replication products were visualized using a Bio-Rad imaging screen K and a Bio-Rad Personal Molecular Imager FX.

### Kinetic analyses of Pol IV and Pol IV-T120P *in vitro* primer extension activity

Kinetic studies using wild type Pol IV or Pol IV-T120P were performed at 25°C in assay buffer (25 mM TrisOAc [pH 7.5], 150 mM KOAc, 10 mM β-mercaptoethanol, 1 mg/ml bovine serum albumin, and 10 mM MgCl_2_). The kinetic parameters (k_pol_, K_d_, and k_pol_/K_d_) for nucleotides were measured as previously described [78]. Briefly, a typical assay was performed by pre-incubating DNA substrate (200 nM) with a 2-fold molar excess of DNA polymerase (400 nM) in assay buffer. Reactions were initiated by adding variable concentrations of nucleotide substrate (1–500 μM). At variable times, 5 μl aliquots of the reaction were removed and immediately quenched by adding an equal volume of 200 mM EDTA. Polymerization reactions were monitored by electrophoresis through 20% sequencing gels as described [79]. Gel images were obtained with a Packard PhosphorImager by using the OptiQuant software supplied by the manufacturer. Product formation was quantified by measuring the ratio of ^32^P-labeled extended and un-extended primer. This ratio was corrected for substrate in the absence of polymerase (zero point). Corrected ratios were multiplied by the concentration of primer/template used in the assay to yield total product. Observed rate constants were obtained using the following equation: y = A*(1-e^kobs*t^)+C, where A is the burst amplitude in product formation, k_obs_ is the observed rate constant of the reaction, t is the time, and C is the end-point in product formation. Data for the dependency of rate constant as a function of nucleotide concentration were fit to the Michaelis-Menten equation: k_obs_ = k_pol_*(dNTP)/(K_d_+[dNTP]), where k_obs_ is the rate constant of the reaction (s^−1^), k_pol_ is the maximal rate constant of polymerization, K_d_ is the apparent dissociation constant for dNTP, and [dNTP] is the concentration of nucleotide substrate.

### Single-molecule primer extension assay

Primer extension by Pol III and Pol IV was observed on single DNA molecules within custom microfluidic flow cells, as previously described [12]. Briefly, primed, single-stranded DNA substrates were derived from 7.2 kb phage M13mp18 DNA (New England Biolabs) end-labeled with digoxigenin and biotin. DNAs were bound to the streptavidin-coated flow cell surface on one end, and to anti-digoxigenin-coupled 2.8 μm-diameter beads on the other. Laminar flow through the flow cell exerted a constant ~3 pN force on the bead, and, by extension, uniformly throughout the tether. Conversion of entropically coiled ssDNA to extended dsDNA by a Pol at this constant force was observed as motion of the bead using dark-field microscopy. Bead positions were tracked by fitting beads to 2D Gaussians, and their motions were converted into DNA synthesis as a function of time. Resolution is determined by thermal fluctuations of the tethered bead (σ ~70 bp) and the choice of exposure time (0.5 s). All experiments were performed in replication buffer (50 mM Hepes-KOH [pH 7.9], 12 mM Mg[OAc]_2_, 80 mM KCl, 0.1 mg/ml BSA, 5 mM DTT) supplemented with 5 nM Pol IIIαεθ, 30 nM β, 15 nM of the τ_3_δδ’ψχ form of the DnaX clamp loader complex, 760 μM dNTPs and 1 mM ATP. Pol IV, Pol IV-T120P, Pol IV^CD^ and Pol IV^CD^-T120P were additionally included at the indicated concentrations. A cutoff of 45 bp/s was used to distinguish Pol III (faster) from Pol IV (slower) events. This cutoff captured ~95% of events in experiments with each polymerase alone. The Pol III replisome components used in the single molecule experiments were purified as previously described: β [80]; α, δ and δ’ [81]; ε and θ [82]; and τ and χψ [83]. The Pol III core αεθ and the clamp loader assembly with stoichiometry τ_3_δδ’χψ were then assembled and purified [83].

## Supporting Information

S1 Fig Steady state levels of mutant Pol IV proteins. Western blot analysis of whole cell lysates of strain MS105 bearing the plasmid expressing the indicated Pol IV protein was performed as described [17,56]. Panels in Blot #2 are from a single exposure of the same membrane. Replicates represent distinct clones (see Table 2). Pol IV^+^ refers to strain MS105 bearing pJH110, while control refers to the MS105 strain bearing pWSK29. Endogenous Pol IV was not detected in this experiment by our anti-Pol IV rabbit polyclonal antibody preparation (see control lane).
(TIF)

S2 Fig Pol IV mutants fail to promote –1 frameshift mutations *in vivo*. Respective frequencies of *lacZ* ^*–*^→*lacZ* ^*+*^ reversion were measured as described previously using strain CC108 [56]. Results shown represent the average of 3 separate determinations ± the range.
(TIFF)

S3 Fig Pol IV mutants fail to confer UV sensitivity. Respective abilities of the 13 plasmid-expressed *dinB* mutations to confer UV sensitivity upon the *dnaN159* Δ(*dinB-yafN*)::*kan* strain (MS116) was measured as described previously [17,48]. The experiment was performed at least 2 times, and representative results are shown. Control refers to strain MS116 bearing pWSK29, while Pol IV^+^ represents strain MS116 bearing pJH110.
(TIFF)

S4 Fig Ability of Pol IV to impede growth of *E*. *coli* MS100 is independent of the oxidized guanine pool. Cultures of strain MS100 bearing the indicated plasmid were serially diluted and spotted onto LB agar plates with or without 0.2% arabinose. For anaerobic growth, plates were placed inside an airtight canister containing palladium catalyst GasPaks (BD Biosciences). Plates were imaged after overnight incubation at 30°C. Results are representative of 2 independent experiments.
(TIF)

S5 Fig Wild type Pol IV expressed form plasmid pRM102 complements the MMS-dependent phenotypes of mutant *dinB* strains. **(A)** The ability of wild type Pol IV expressed from pRM102 to complement MMS sensitivity of the indicated *dinB* strains, or **(B)** their respective inabilities to suppress MMS-induced mutagenesis are shown. Results in panel A are representative of 4 independent experiments, while those in panel B are the average of 2 independent experiments ± the range.
(TIFF)

S6 Fig Pol IV residues involved in functional and/or physical interactions with UmuD, RecA or Pol III. Positions of Pol IV mutations identified by Benson *et al*. [61] that abrogated lethality caused by overproduced levels of Pol IV in the *dnaE915* strain are represented on the *in silico* model of the Pol IIIαεθ-β clamp-DNA complex in either **(A)** ribbon or **(B)** surface views. Residue V7 and F292 of Pol IV are shown in black, while T120 is in orange. Positions of Pol IV residues identified by Godoy *et al*. [64] demonstrated to interact with UmuD are represented on the *in silico* model of the Pol IIIαεθ-β clamp-DNA complex in either **(C)** ribbon or **(D)** surface views. Residues P166, F172 and L176 of Pol IV are shown in pink, while T120 is in orange.
(TIFF)

S1 Table Pol IV mutants are unable to impede growth of the *dnaN159* strain. (DOCX)

S2 Table The ability of Pol IV to impede growth of the *dnaN159* strain is independent of aerobic growth. (DOCX)

S3 Table Oligonucleotides used in this study. (DOCX)
