# Supplementary figures and images for: A Genetic Selection for dinB Mutants Reveals an Interaction between DNA Polymerase IV and the Replicative Polymerase That Is Required for Translesion Synthesis
Source: PLoS Genet. 2015 Sep 9;11(9):e1005507. doi: 10.1371/journal.pgen.1005507 (PMC4564189; doi:10.1371/journal.pgen.1005507)

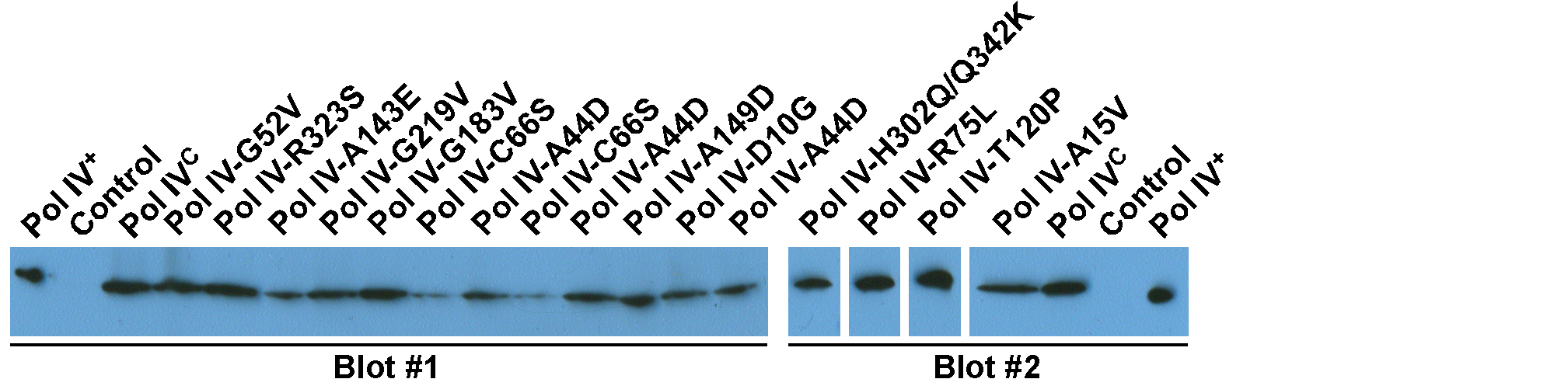

Supplement: S1 Fig — Western blot analysis of whole cell lysates of strain MS105 bearing the plasmid expressing the indicated Pol IV protein was performed as described [17,56]. Panels in Blot #2 are from a single exposure of the same membrane. Replicates represent distinct clones (see Table 2). Pol IV+ refers to strain MS105 bearing pJH110, while control refers to the MS105 strain bearing pWSK29. Endogenous Pol IV was not detected in this experiment by our anti-Pol IV rabbit polyclonal antibody preparation (see control lane). (TIF) [file pgen.1005507.s001.tif]

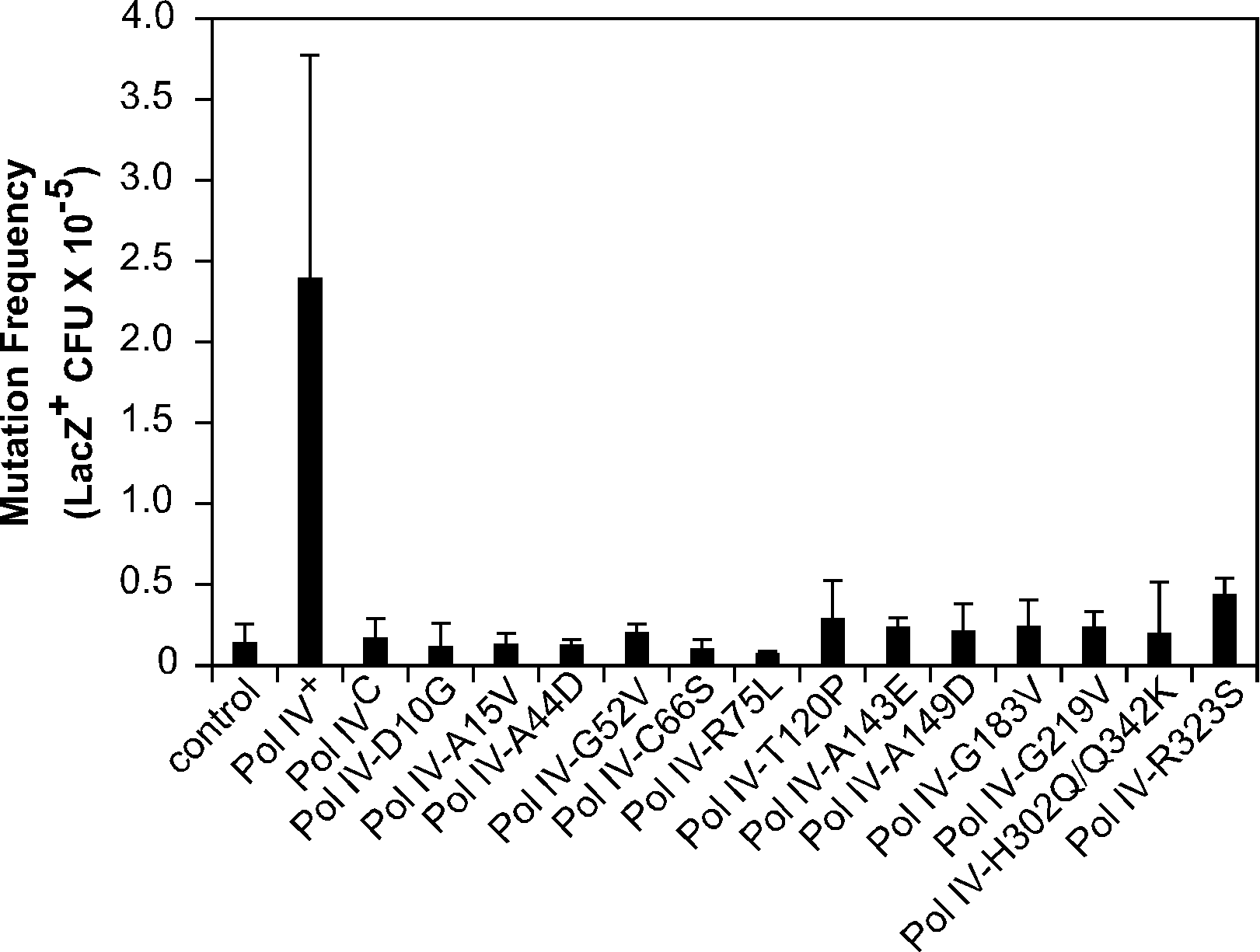

Supplement: S2 Fig — Respective frequencies of lacZ –→lacZ + reversion were measured as described previously using strain CC108 [56]. Results shown represent the average of 3 separate determinations ± the range. (TIFF) [file pgen.1005507.s002.tiff]

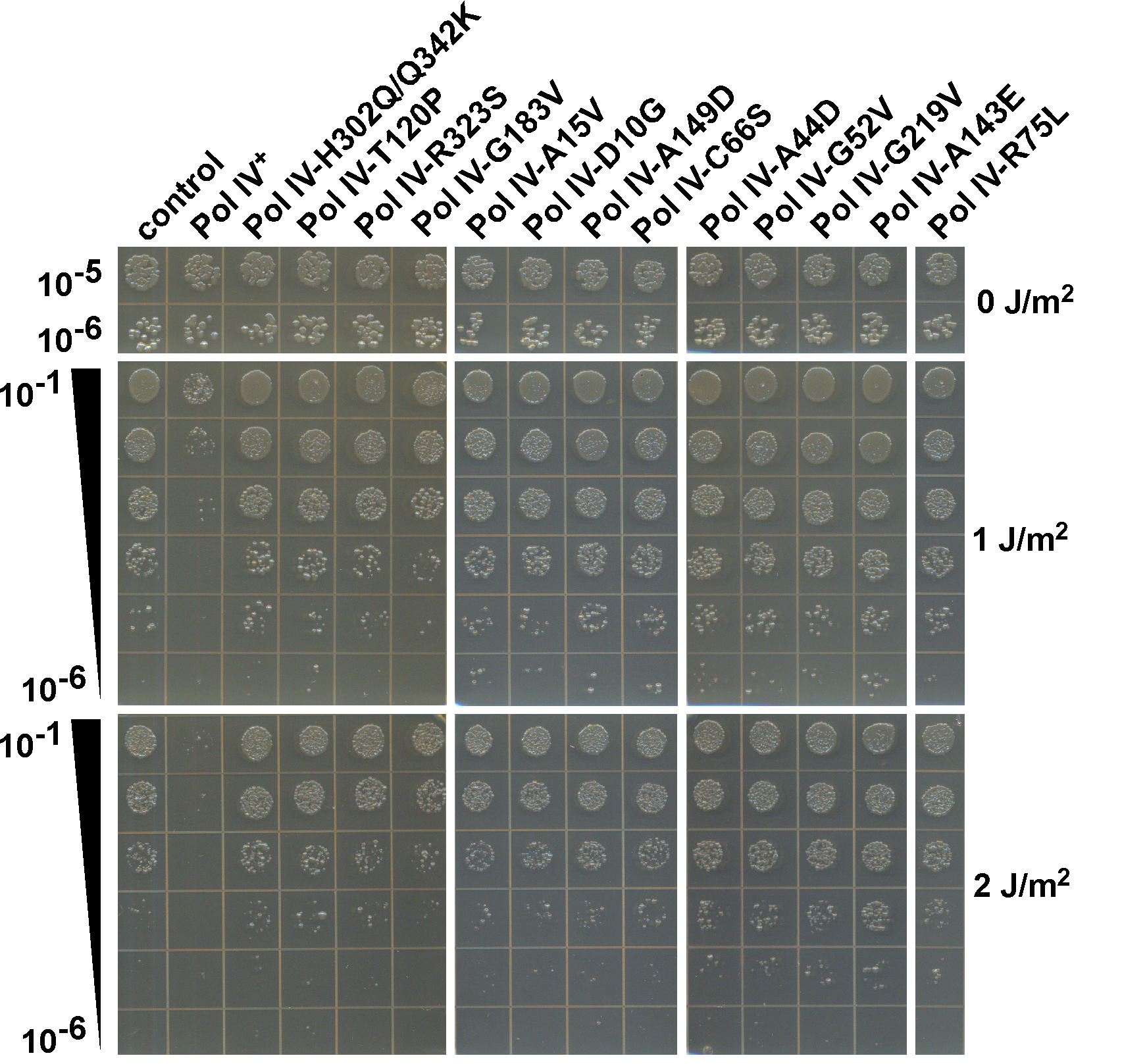

Supplement: S3 Fig — Respective abilities of the 13 plasmid-expressed dinB mutations to confer UV sensitivity upon the dnaN159 Δ(dinB-yafN)::kan strain (MS116) was measured as described previously [17,48]. The experiment was performed at least 2 times, and representative results are shown. Control refers to strain MS116 bearing pWSK29, while Pol IV+ represents strain MS116 bearing pJH110. (TIFF) [file pgen.1005507.s003.tiff]

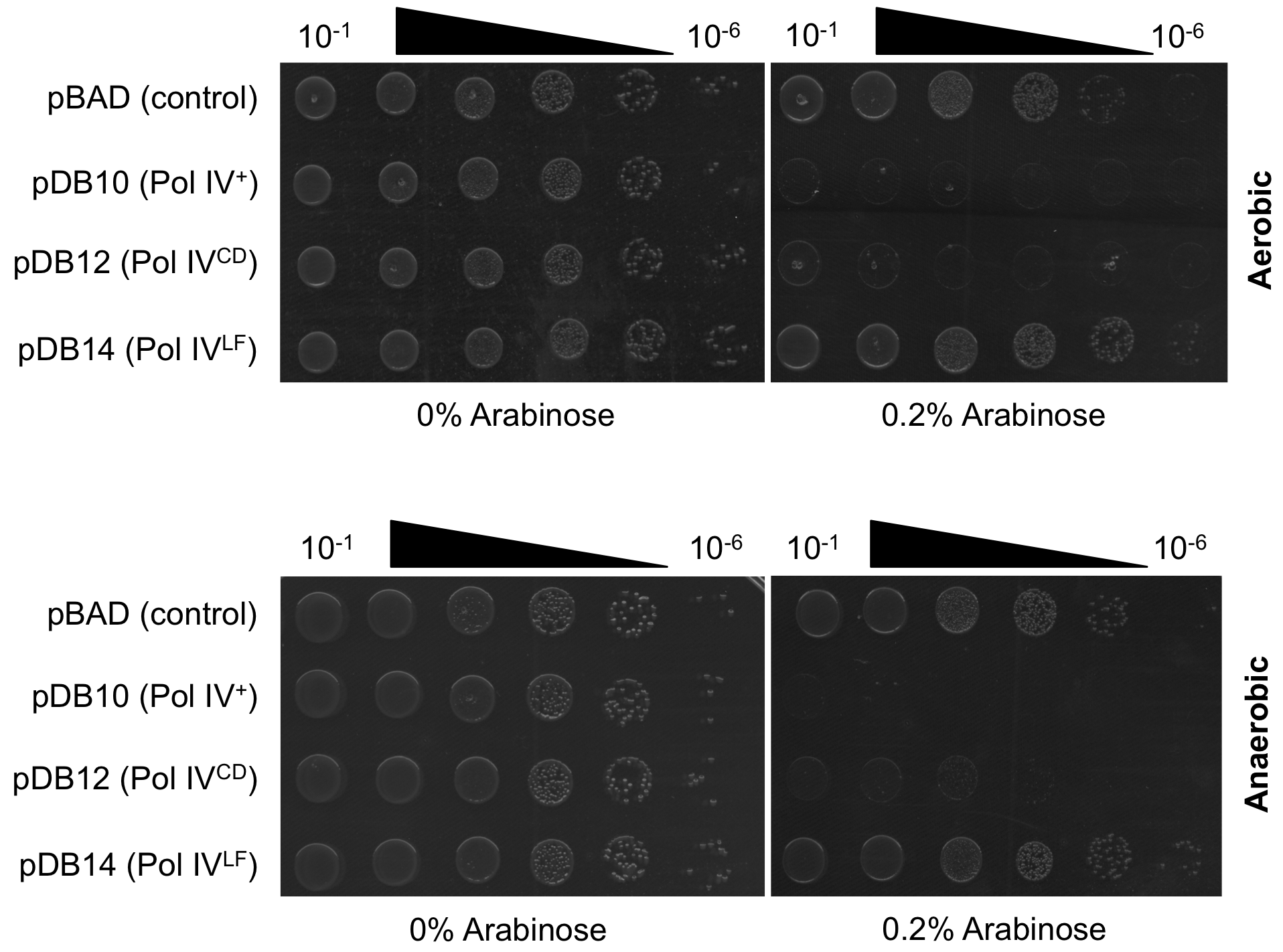

Supplement: S4 Fig — Cultures of strain MS100 bearing the indicated plasmid were serially diluted and spotted onto LB agar plates with or without 0.2% arabinose. For anaerobic growth, plates were placed inside an airtight canister containing palladium catalyst GasPaks (BD Biosciences). Plates were imaged after overnight incubation at 30°C. Results are representative of 2 independent experiments. (TIF) [file pgen.1005507.s004.tif]

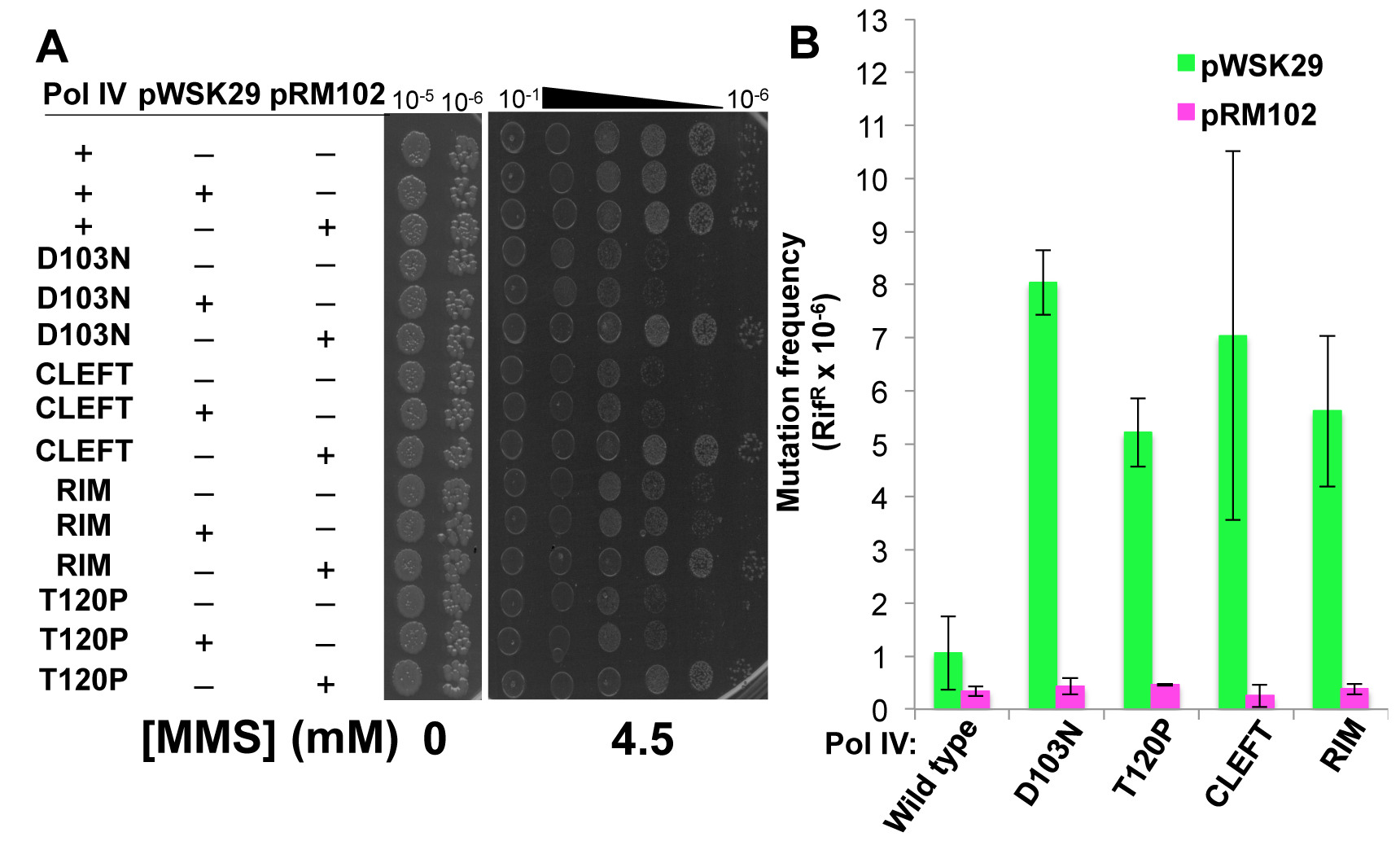

Supplement: S5 Fig — (A) The ability of wild type Pol IV expressed from pRM102 to complement MMS sensitivity of the indicated dinB strains, or (B) their respective inabilities to suppress MMS-induced mutagenesis are shown. Results in panel A are representative of 4 independent experiments, while those in panel B are the average of 2 independent experiments ± the range. (TIFF) [file pgen.1005507.s005.tiff]

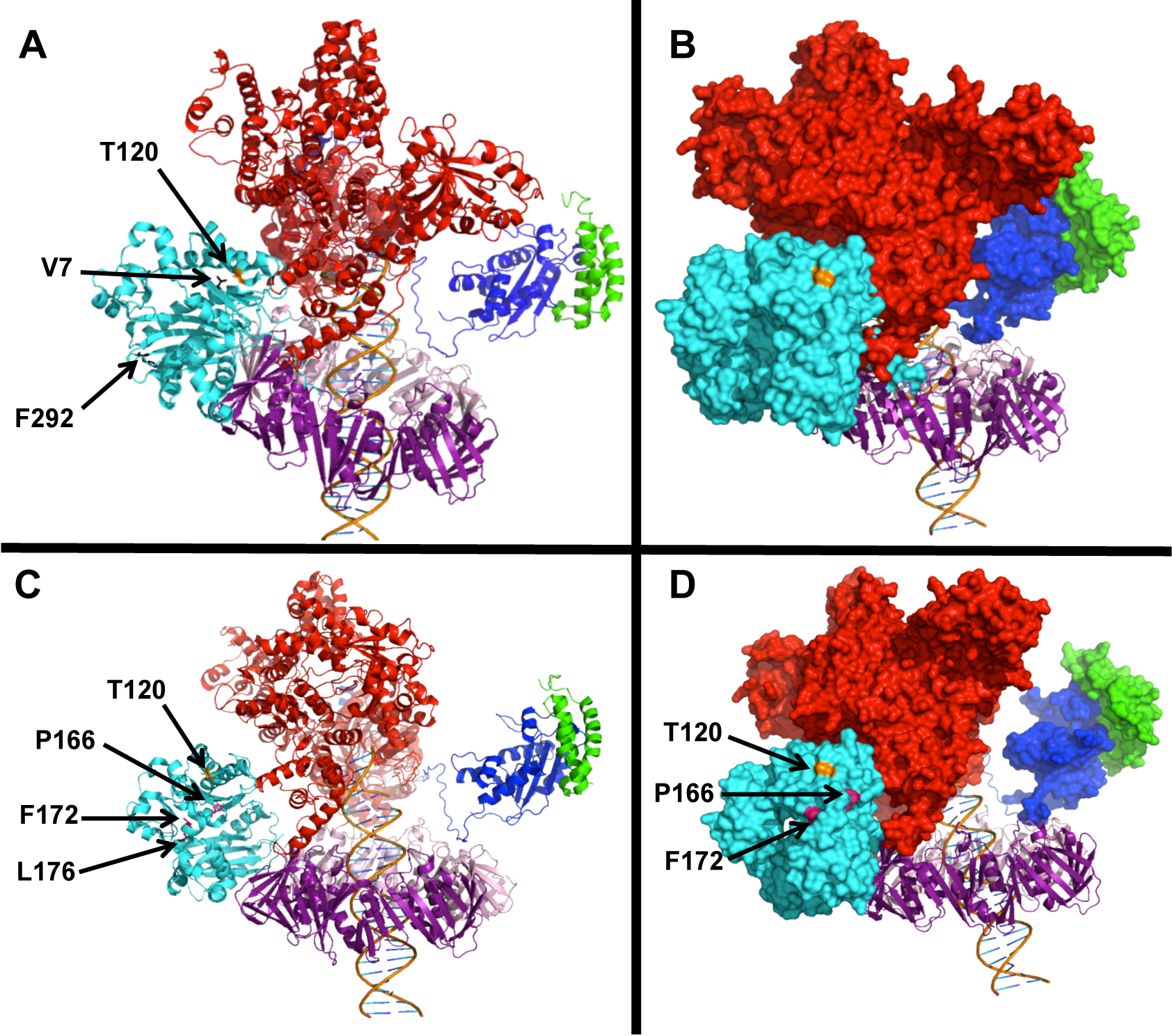

Supplement: S6 Fig — Positions of Pol IV mutations identified by Benson et al. [61] that abrogated lethality caused by overproduced levels of Pol IV in the dnaE915 strain are represented on the in silico model of the Pol IIIαεθ-β clamp-DNA complex in either (A) ribbon or (B) surface views. Residue V7 and F292 of Pol IV are shown in black, while T120 is in orange. Positions of Pol IV residues identified by Godoy et al. [64] demonstrated to interact with UmuD are represented on the in silico model of the Pol IIIαεθ-β clamp-DNA complex in either (C) ribbon or (D) surface views. Residues P166, F172 and L176 of Pol IV are shown in pink, while T120 is in orange. (TIFF) [file pgen.1005507.s006.tiff]
