# Supplementary material for: A Genetic Selection for dinB Mutants Reveals an Interaction between DNA Polymerase IV and the Replicative Polymerase That Is Required for Translesion Synthesis
Source: PLoS Genet. 2015 Sep 9;11(9):e1005507. doi: 10.1371/journal.pgen.1005507 (PMC4564189; doi:10.1371/journal.pgen.1005507)
Supplement: S1 Table — (DOCX) [file pgen.1005507.s007.docx]

**S1 Table. Pol IV mutants are unable to impede growth of the *dnaN159* strain.**

| **Plasmid** | **Pol IV protein** | **Transformation efficiency**  **(relative to pWSK29 vector control) *^a^*** | |
| --- | --- | --- | --- |
|  |  | **MS104**  **(*dnaN^+^ lexA51*[Def])** | **MS105**  **(*dnaN159 lexA51*[Def])** |
| pWSK29 | NA ***^b^***  (control) | 5.87 (±2.12) x 10^3^  (≡1.0) | 6.11 (±1.79) x 10^3^  (≡1.0) |
| pRM102 | Pol IV^+^ | 4.22 (±0.46) x 10^3^  (0.72) | <6.6  (<0.001) |
| pJH110 ***^c^*** | Pol IV^+^ | 3.59 (±0.08) x 10^3^  (0.61) | <6.0  (<0.001) |
| pJH101 | Pol IV^R^ | 3.30 (±0.13) x 10^3^  (0.56) | 2.06 (±0.03) x 10^3^  (0.34) |
| pJH102 | Pol IV^C^ | 3.24 (±0.33) x 10^3^  (0.55) | 3.98 (±0.11) x 10^3^  (0.65) |
| pJH100 | Pol IV-D103N | 3.27 (±1.39) x 10^3^  (0.56) | <8.3  (<0.001) |
| pJH111 | Pol IV-D10G | 5.56 (±1.32) x 10^3^  (0.95) | 6.31 (±3.07) x 10^3^  (1.03) |
| pJH150 | Pol IV-A15V | 3.33 (±0.61) x 10^3^  (0.57) | 3.10 (±0.03) x 10^3^  (0.51) |
| pJH152 | Pol IV-A44D | 5.37 (±0.60) x 10^3^  (0.91) | 5.84 (±0.38) x 10^3^  (0.96) |
| pJH154 | Pol IV-G52V | 4.77 (±0.29) x 10^3^  (0.81) | 5.14 (±0.44) x 10^3^  (0.84) |
| pJH112 | Pol IV-C66S | 4.73 (±0.20) x 10^3^  (0.81) | 3.87 (±2.14) x 10^3^  (0.63) |
| pJH113 | Pol IV-R75L | 3.32 (±0.32) x 10^3^  (0.57) | 5.11 (±0.35) x 10^3^  (0.84) |
| pJH114 | Pol IV-T120P | 5.10 (±0.08) x 10^3^  (0.87) | 4.74 (±0.07) x 10^3^  (0.78) |
| pJH156 | Pol IV-A143E | 4.19 (±0.45) x 10^3^  (0.71) | 0.85 (±0.47) x 10^3^  (0.14) |
| pJH115 | Pol IV-A149D | 6.48 (±0.91) x 10^3^  (1.10) | 5.92 (±0.51) x 10^3^  (0.97) |
| pJH116 | Pol IV-G183V | 5.15 (±0.25) x 10^3^  (0.88) | 3.12 (±0.08) x 10^3^  (0.51) |
| pJH157 | Pol IV-G219V | 6.37 (±0.09) x 10^3^  (1.08) | 4.40 (±0.14) x 10^3^  (0.72) |
| pJH117 | Pol IV-H302Q/Q342K | 6.40 (±0.05) x 10^3^  (1.09) | 5.20 (±0.21) x 10^3^  (0.85) |
| pJH118 | Pol IV-R323S | 4.80 (±0.03) x 10^3^  (0.82) | 4.27 (±0.03) x 10^3^  (0.70) |

***^a^*** Transformation frequencies represent an average of 2-4 independent determinations, ± range. Values in parentheses represent transformation efficiency of that strain relative to the pWSK29 control, which was set equal to 1.0 (≡1.0).

***^b^*** NA, not applicable; pWSK29 does not express Pol IV.

***^c^*** Plasmid pJH110 is identical to pRM102 except for the NdeI restriction site overlapping the N-terminal methionine to facilitate subcloning from pWSK29 into pET11a (see *Materials and Methods*).
