# Supplementary material for: A Genetic Selection for dinB Mutants Reveals an Interaction between DNA Polymerase IV and the Replicative Polymerase That Is Required for Translesion Synthesis
Source: PLoS Genet. 2015 Sep 9;11(9):e1005507. doi: 10.1371/journal.pgen.1005507 (PMC4564189; doi:10.1371/journal.pgen.1005507)
Supplement: S2 Table — (DOCX) [file pgen.1005507.s008.docx]

**S2 Table. The ability of Pol IV to impede growth of the *dnaN159* strain is independent of aerobic growth.**

| **Plasmid** | **Transformation efficiency *^a^*** | | | |
| --- | --- | --- | --- | --- |
|  | **MS104 (*dnaN^+^ lexA51*[Def])** | | **MS105 (*dnaN159 lexA51*[Def])** | |
|  | **Aerobic** | **Anaerobic** | **Aerobic** | **Anaerobic** |
| pWSK29  (control) | 9.49 (±0.01) x 10^3^  (≡1.0) | 9.60 (±0.12) x 10^3^  (≡1.0) | 9.03 (±0.59) x 10^3^  (≡1.0) | 8.46 (±0.26) x 10^3^  (≡1.0) |
| pRM102  (Pol IV^+^) | 6.34 (±0.69) x 10^3^  (0.67) | 5.96 (±0.40) x 10^3^  (0.62) | <10 (±0.00)  (<0.001) | <10 (±0.00)  (<0.001) |

***^a^*** Strains and plasmids are described in Table 1. Values shown represent the average of 4 independent determinations ± standard deviation. Values in parentheses represent transformation efficiencies relative to the frequency observed for the pWSK29 control, which was set equal to 1.0 (≡1.0). Anaerobic growth conditions were verified using the ∆*mutT* strain JW0097, which displays a spontaneous mutator phenotype when grown aerobically, but not when grown anaerobically due to incorporation of 8-oxo-dG into nascent DNA, leading to G→T transversions [1]. Under conditions used for this experiment (see *Materials and Methods*), the spontaneous frequency of rifampicin resistance for strain JW0097 grown aerobically was 3.56 (2.30-5.87) x 10^-6^, compared to 4.17 (2.88-4.58) x10^-8^ when grown anaerobically. These values for the ∆*mutT* strain represent the median from 10 independent determinations ± 95% confidence intervals, which are in parentheses.
