# Supplementary material for: A Genetic Selection for dinB Mutants Reveals an Interaction between DNA Polymerase IV and the Replicative Polymerase That Is Required for Translesion Synthesis
Source: PLoS Genet. 2015 Sep 9;11(9):e1005507. doi: 10.1371/journal.pgen.1005507 (PMC4564189; doi:10.1371/journal.pgen.1005507)
Supplement: S3 Table — (DOCX) [file pgen.1005507.s009.docx]

**S3 Table. Oligonucleotides used in this study.**

| **Oligonucleotide** | **Nucleotide sequence (5’→3’)** | **Source** |
| --- | --- | --- |
| *dinB*-NdeI-Top | GTTGATAGGTGACATATGCGTAAAATCATTCATGTG | IDT |
| *dinB*-NdeI-Bottom | CACATGAATGATTTTACGCATATGTCACCTCTCAAC | IDT |
| *dinB*-D10G-Forward | C ATTCATGTGGATATGGGCTGCTTTTTCGCC | IDT |
| *dinB*-D10G-Reverse | GGCGAAAAAGCAGCCCATATCCACATGAATG | IDT |
| *dinB*-C66S-Forward | GGCGCTCAAATTAAGCCCACATCTCACC | IDT |
| *dinB*-C66S-Reverse | GGTGAGATGTGGGCTTAATTTGAGCGCC | IDT |
| *dinB*-R75L-Forward | CCTTGCTTCCGGGGCTCTTTGACGCCTAC | IDT |
| *dinB*-R75L-Reverse | GTAGGCGTCAAAGAGCCCCGGAAGCAAGG | IDT |
| *dinB*-T120P-Forward | GCCACGGTTCTGCGCCCCTCATCGCCCAGG | IDT |
| *dinB*-T120P-Reverse | CCTGGGCGATGAGGGGCGCAGAACCGTGGC | IDT |
| *dinB*-G183V-Forward | GCAAAAATCCCCGTCGTCGGCAAAGTCTC | IDT |
| *dinB*-G183V-Reverse | GAGACTTTGCCGACGACGGGGATTTTTGC | IDT |
| 30-mer | TAGTTAGCGTTTCGATCTAAAGTTTTGTCG | IDT |
| 100-mer ***^a^*** | AATCCCATACAGAAAATTCATTTACTAACGTCTGGAAACTcgacaaaactttagatcgaaacgctaactaTGAGGGGTGTCTGTGGAATGCTACAGGCGT | IDT |
| 13-mer | TCGCAGCCGTCCA | Operon |
| 20G | AAACCT<G>TGGACGGCTGCGA | Operon |
| 20MeG | AAACCT<*O^6^*-mdG>TGGACGGCTGCGA | Operon |
| 20A | AAACCT<A>TGGACGGCTGCGA | Operon |
| 20MeA | AAACCT<3d-medA>TGGACGGCTGCGA | IDT |
| 20Sp | AAACCT<Sp>TGGACGGCTGCGA | Operon |
| P1 | CTGGTGCAAAAGCTGGATAAGCAGCAGGTGC | IDT |
| P4 | GACCGATTTTTCAGCGAGAATTCGATGC | IDT |
| MKS028 | GCATGGGGATAAAGTGGTGC | IDT |
| MKS037 | GCAAGAATGTGAATAAAGGCCGG | IDT |
| MKS039 | GGCATGCTGAATCTTTACGC | IDT |
| MKS040 | GCGTCCATTGCCACGGTTCTGC | IDT |
| MKS042 | GGCAAAACTGGAAGCGATGG | IDT |
| MKS043 | CCCAGGAGCACGTCTGGC | IDT |
| MKS045 | GCGTGTAATTTCCTCTAATCTTGC | IDT |
| MKS046 | CGCAACTGTGGCGGAAATGG | IDT |
| MKS051 | CCTTGCTTCCGGGGCGCTTTGACGCCTAC | IDT |
| MKS055 | CCAGGGATTACGCGTGC | IDT |
| MKS056 | GGTAAGCGCAATGGCGG | IDT |

***^a^*** Sequence in lower case represents the homology to the 30-mer primer.
